# Opposite Carcinogenic Effects of Circadian Clock Gene *BMAL1*

**DOI:** 10.1038/s41598-018-34433-4

**Published:** 2018-10-30

**Authors:** Tuba Korkmaz, Fatih Aygenli, Handan Emisoglu, Gozde Ozcelik, Asena Canturk, Secil Yilmaz, Nuri Ozturk

**Affiliations:** 1 Department of Molecular Biology and Genetics, Gebze Technical University, Gebze, Kocaeli Turkey; 2 Genome and Stem Cell Center (GENKOK), Erciyes University, Kayseri, Turkey

## Abstract

The circadian clock confers daily rhythmicity on many biochemical and physiological functions and its disruption is associated with increased risks of developing obesity, diabetes, heart disease and cancer. Although, there are studies on the role of *Bmal1* in carcinogenesis using germline, conditional or tissue-specific knockouts, it is still not well understood how *BMAL1* gene affects cancer-related biological events at the molecular level. We, therefore, took an *in vitro* approach to understand the contribution of *BMAL1* in this molecular mechanism using human breast epithelial cell lines by knocking out *BMAL1* gene with CRISPR technology. We preferred epithelial cells over fibroblasts as the most of cancers originate from epithelial cells. After obtaining *BMAL1* knockouts by targeting the gene at two different sites from non-tumorigenic MCF10A and invasive tumorigenic MDA-MB-231 cells, we analysed apoptosis and invasion properties of the cell lines as representative events in tumor development. *BMAL1* disruption sensitized both cell lines to a bulky-DNA adduct forming agent (cisplatin) and a double-strand break-inducing agent (doxorubicin), while it enhanced the invasive properties of MDA-MB-231 cells. These results show that the disruption of clock genes may have opposing carcinogenic effects.

## Introduction

The circadian rhythms are the daily oscillations in behavioural, physiological, and metabolic processes. In mammalian cells, these rhythms are generated by an endogenous self-sustaining molecular clock based on a transcription-translation feedback loop (TTFL). On the positive or inductive limb of this TTFL, the transcription factors BMAL1 (encoded by *ARNTL* gene, *Aryl Hydrocarbon Receptor Nuclear Translocator Like*) and CLOCK transactivate the negative or repressive limb factors, *Cryptochrome* (*CRY1* and *CRY2*) and *Period* (*PER1* and *PER2*), as well as circadian clock-regulated genes (CCGs). Translated products of the negative limb then translocate into the nucleus after being detained in the cytoplasm, and interact with the positive factors to inhibit their transcriptional activity by protein–protein interactions^1^. This transactivation and inhibition by positive and negative feedback generates an oscillation in the expression of CCGs, including the *CRY* and *PER* genes^2,3^. However, the period of this oscillation is tuned up to ~24 hours by secondary loops and post-translational modifications^4–6^. It is thought that 10% of the transcriptome and 20% of the proteome are regulated in a circadian manner and the percentage of rhythmic transcriptome or proteome varies from tissue to tissue, which indicates that the circadian clock is important for the homeostasis of the cellular environment^7,8^. Moreover, Zhang *et al*. found that 43% of all protein-coding genes showed circadian oscillations in transcription somewhere in the body, largely in an organ-specific manner^8^. Additionally, the functionality of the circadian clock is attenuated or disrupted with age^9,10^. As a consequence, the disruption of the circadian clock has been associated with a variety of physio-pathological states, ranging from metabolic disorder to cancer.

Epidemiological data suggest that individuals with occupational circadian clock disruption have an increased risk of cancer^11,12^. However, these studies do not differentiate whether that increased risk is caused by the disruption of the clock or its associated effects such as a change in lifestyle, and thus they have not revealed any mechanistic insights. Therefore, animal models have been used for understanding the mechanistic relationship between the circadian clock and cancer. These models have been generated to study either spontaneous or induced carcinogenesis. Testing the relationship between the circadian clock genes and tumorigenesis started with the germline knockouts of the circadian clock genes. In an early animal model, *Per2* mutant mice were found to be predisposed to spontaneous and irradiation-induced cancers^13^. In another study, loss of *Per* genes (*Per1* or *Per2*) did not show a significant effect on spontaneous tumor onset in non-irradiated mice or after irradiation^14^. In respect to the other component of the negative limb of TTFL, *Cry* double knockout (*Cry* DKO) mice were found to be indistinguishable from wild-type mice in respect to spontaneous and irradiation-induced cancer^15^. Thus, to exclude the possibility that a small increase in cancer risk was missed in previous studies, *Cry* mutations were combined with a *p53* null mutation^16^. Tumor suppressor *p53* (also known as *TP53*) gene is the most frequently mutated gene in human cancers. P53 protein is activated upon the formation of some signals such as DNA damage and governs some anti-proliferative transcriptional program to handle the stress conditions or in the case of long duration of insults, induces apoptotic cell death program to reduce the accumulation of carcinogenic mutations^17^. On its own, *p53* mutations predispose mice to lymphoma by the age of 6 months^18^. Although the authors expected to see an increased cancer incidence on a *p53* null background, *Cry* deletion in this context increased the tumor free life-span as much as 1.5-fold^16^. Using fibroblasts isolated from the skin of *Cry:p53* and *p53* null mice, they showed that *Cry* deletion on the *p53* null background sensitized the cells to bulky-DNA adduct-induced apoptosis through circadian clock-regulated Egr1-mediated p73 induction^19,20^. On the other hand, it was later reported that there is an increased tumor burden in *Cry2* KO mice^21^ in opposite to *Cry* DKO mice.

When the positive limb components of the TTFL were knocked out in mice, different phenotypes were observed in respect to tumorigenesis. *Clock* knockout mice did not have an increased incidence of cancer^22,23^ while whole-body *Bmal1* knockout mice had an increased tumor burden^24^. A study by Lee *et al*. found that virtually all lines of mice (including *Cry* DKO, *Clock*Δ19/Δ19, and lacking one copy of *Bmal1*) which they examined had enhanced tumorigenesis under basal and irradiated conditions^25^. Germline *Bmal1* null mice, and to a lesser extend *Clock* null mice, exhibited early aging phenotypes^26^, and this problem was bypassed with the generation of a conditional *Bmal1* knockout mouse model which lacked BMAL1 protein only during adult life^27^.

In summary, considering the whole-body knockouts of the circadian clock genes, there are different outputs in respect to the relationship between the genetic disruption of the circadian clock and cancer risk. This spectrum of the different results with the circadian clock gene knockouts and tumorigenesis suggests that more studies are needed including *in vitro* models such as genetic modification of isolated cell line in order to pinpoint the relationship between circadian clock genes and other pathways including the ones important in carcinogenesis and to study molecular events associated with carcinogenesis.

In this study, we investigated the relationship between *BMAL1* knockout mutation and carcinogenesis at molecular level using cell lines. Although previous studies investigated mouse embryonic fibroblasts from *Bmal1* knockout mice, no significant change in DNA repair or DNA damage responses were reported^28^. However, fibroblasts are not the most appropriate model to study carcinogenic events because most tumors originate from epithelial cells rather than fibroblasts. In order to investigate the molecular events, cell lines are isolated from animal models mostly in the form of fibroblasts, and this whole process takes a long time. However, the recent development of novel and highly efficient DNA editing techniques such as CRISPR/Cas9 allows for fast, inexpensive, and precise gene editing in cell lines^29^. Therefore, it is now possible to dissect the effect of any gene of interest by genome editing using different mammalian cell lines, which are typically used to study carcinogenic events as models. With the use of novel genome editing methods, we thought that we can form a platform to study the effect of *Bmal1* gene deletion at cellular and mechanistic level which may help us with investigating the relationship between *Bmal1* gene disruption and the steps of multifactorial and multistage events in cancer development using non-tumorigenic MCF10A and invasive tumorigenic MDA-MB-231 cell lines.

## Results

In contrast to other circadian clock gene knockouts, *Bmal1* is the only gene whose deletion alone leads to complete loss of rhythmicity^30^. Although, there are studies on the role of *Bmal1* in carcinogenesis using germline, conditional or tissue specific knockouts, we still need to understand how *BMAL1* contributes to carcinogenesis process at the molecular level. Therefore, we knocked out the *BMAL1* gene in a nearly normal cell line (MCF10A) and an invasive cell line (MDA-MB-231) to study two cancer-related pathways, apoptosis and invasion. We exploited CRISPR by targeting two different genomic sites separately in *BMAL1* gene to obtain *BMAL1* knockouts in these cell lines. We first investigated the effect of *BMAL1* gene deletion on the apoptosis in response to genotoxic agents in MCF10A cells, which express wild-type *p53* and are not transformed. To assess the effect of the *BMAL1* knockout mutation on invasive potential, we then used the invasive MDA-MB-231 cell line. However, we later extended our analysis on apoptosis by using MDA-MB-231 to see whether the observed apoptotic response in untransformed MCF10A is also preserved in a transformed and invasive cancer cell line.

### Knockout of *BMAL1* in cell lines

**Figure 1:**
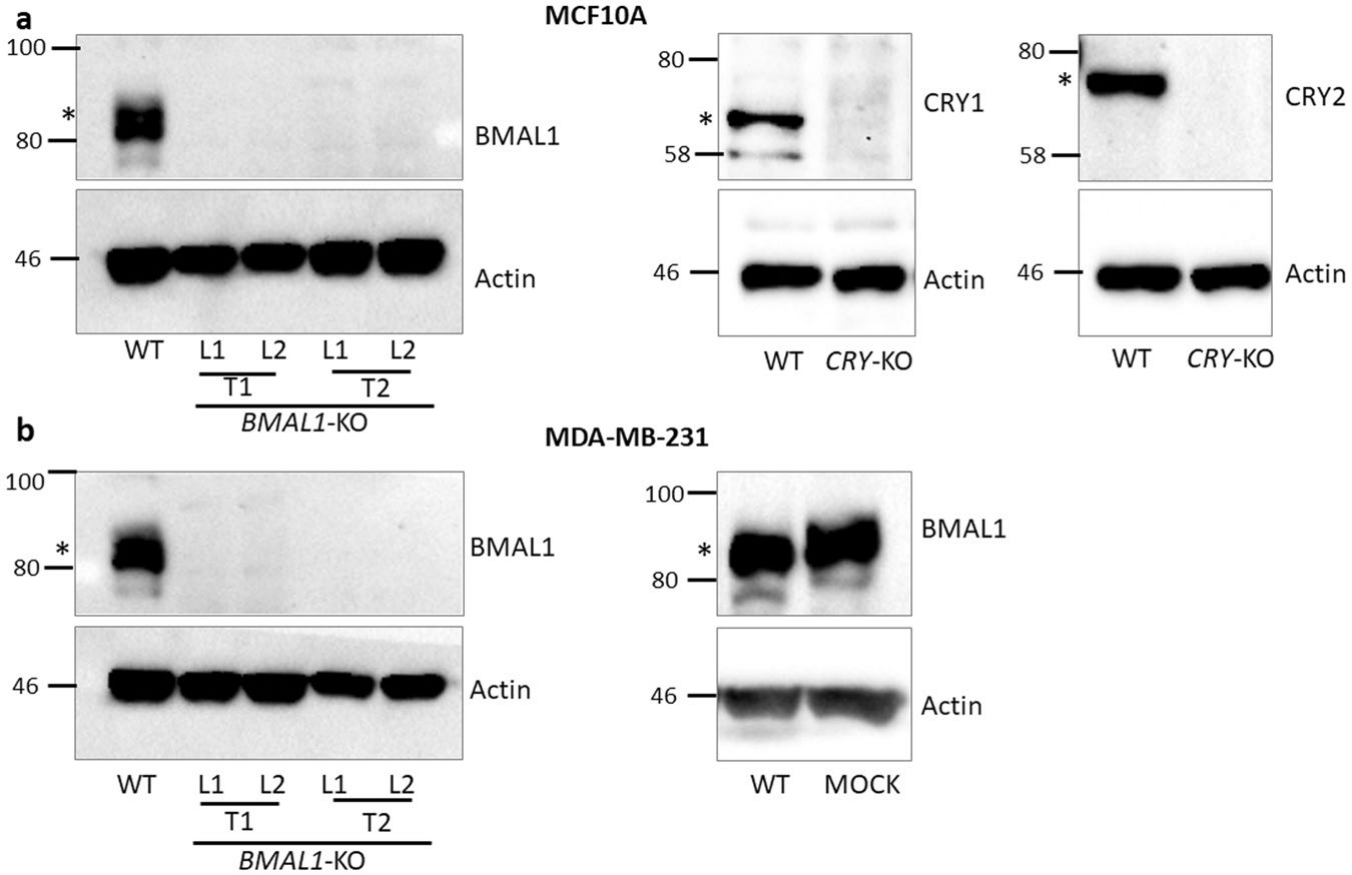
Confirmation of *BMAL1* knockouts. The *BMAL1* gene was knocked out using the LentiCRISPRv2 system by targeting the gene separately at two different locations using T1 and T2 sgRNAs to eliminate non-specific off-target effects. *CRY1* and *CRY2* (*CRY*-DKO) were also knocked out in both cell lines as a control (MDA-MB-231 *CRY*-DKO data are presented in Supplementary Fig. S4). (**a**) Immunoblot of BMAL1, CRY1, and CRY2 proteins in the MCF10A clones confirmed that the knockouts were successful. *CRY*-DKO was used a control for the apoptosis experiments because *CRY* did not affect apoptosis in the presence of wild-type p53. (**b**) MDA-MB-231 *BMAL1* knockouts were obtained using the same protocol as for MCF10A, and the knockouts were confirmed with BMAL1 immunoblotting. Because MDA-MB-231 cells have mutant *p53*, *CRY* mutation amplified apoptosis in response to cisplatin treatment, as expected, which is in agreement with our previous publication (see Supplementary Fig. S4). We obtained a mock cell line of MDA-MB-231, which was infected with *BMAL1-*targeting lentivirus, but escaped the knockout process as confirmed by the presence of the BMAL1 protein. Actin was blotted as a loading control. T1 and T2 represent two separate sgRNAs for *BMAL1* gene. L1 and L2 are subclone lines originating from T1 or T2 targeting. WT represents parental cell lines, and * indicates the specific signal for circadian clock proteins, which were blotted. Numbers and marks on the left of each figure indicate the positions of the corresponding molecular size markers in kDa. Full-length blots are presented in Supplementary Fig. S8.

We used the CRISPR design method^31^ and the CRISPR Design Tool^29,32^ to select efficient sgRNAs and construct plasmids to target the genes. MCF10A and MDA-MB-231 cell lines were infected with lentivirus particles encoding wild-type Cas9 and the sgRNAs. *BMAL1* was targeted at two different locations with minimal off-target binding, and two knockout subclones for each target sgRNA were selected for further studies. Knockouts were screened with immunoblotting using a specific anti-BMAL1 antibody against the C-terminus of the protein (Fig. 1). We obtained two subclones for each *BMAL1* knockout, and a *CRY*-DKO mutant control for MCF10A and a mock control for MDA-MB-231, which were transfected with *BMAL1-*targeting lentivirus particles but escaped the knockout process. In Fig. 1, T1 and T2 indicate that *BMAL1* was disrupted by two different target (T) sgRNAs, and Line 1 (L1) and line 2 (L2) are different subclones for T1 (sgRNA) or different subclones of T2 (sgRNA). The same nomenclature was also used for MDA-MB-231 cell line knockouts. *CRY*-DKO was used as a control for MCF10A in addition to the parental MCF10A line. Although we obtained a *CRY*-DKO of MDA-MB-231, it showed sensitivity to cisplatin-induced apoptosis because of a *p53* mutation in this cell line, which was in agreement with our previous findings^16^. This was also explained by experimental findings below. Therefore, we used the mock control in addition to the parental MDA-MB-231 cell line to compare the knockouts. We confirmed the mutations using the T7 Endonuclease assay (Supplementary Fig. S1). In order to understand the nature of the mutations induced by CRISPR in *BMAL1* knockouts, we sequenced PCR products from cDNAs or gDNAs for T1 and T2 induced knockouts, respectively, and analysed the Sanger sequencing results to detect the indels by CRISP-ID method^33^. We found that all mutations in knockouts generated early stop codons before the end of the basic helix loop helix (bHLH) domain (Supplementary Fig. S1). These truncated parts of BMAL1 protein are far from being a functional BMAL1 because they do not have a full bHLH domain as well as PAS domains which are important for dimerization^34^. Moreover, the truncations with T1 sgRNA generated proteins (stops at codon 58–64) just before the start of the first helix (which starts at codon 62) which would not fold into a functional helix structure.

### The effect of *BMAL1* knockout mutation on apoptosis induced by UV-mimetic cisplatin

**Figure 2:**
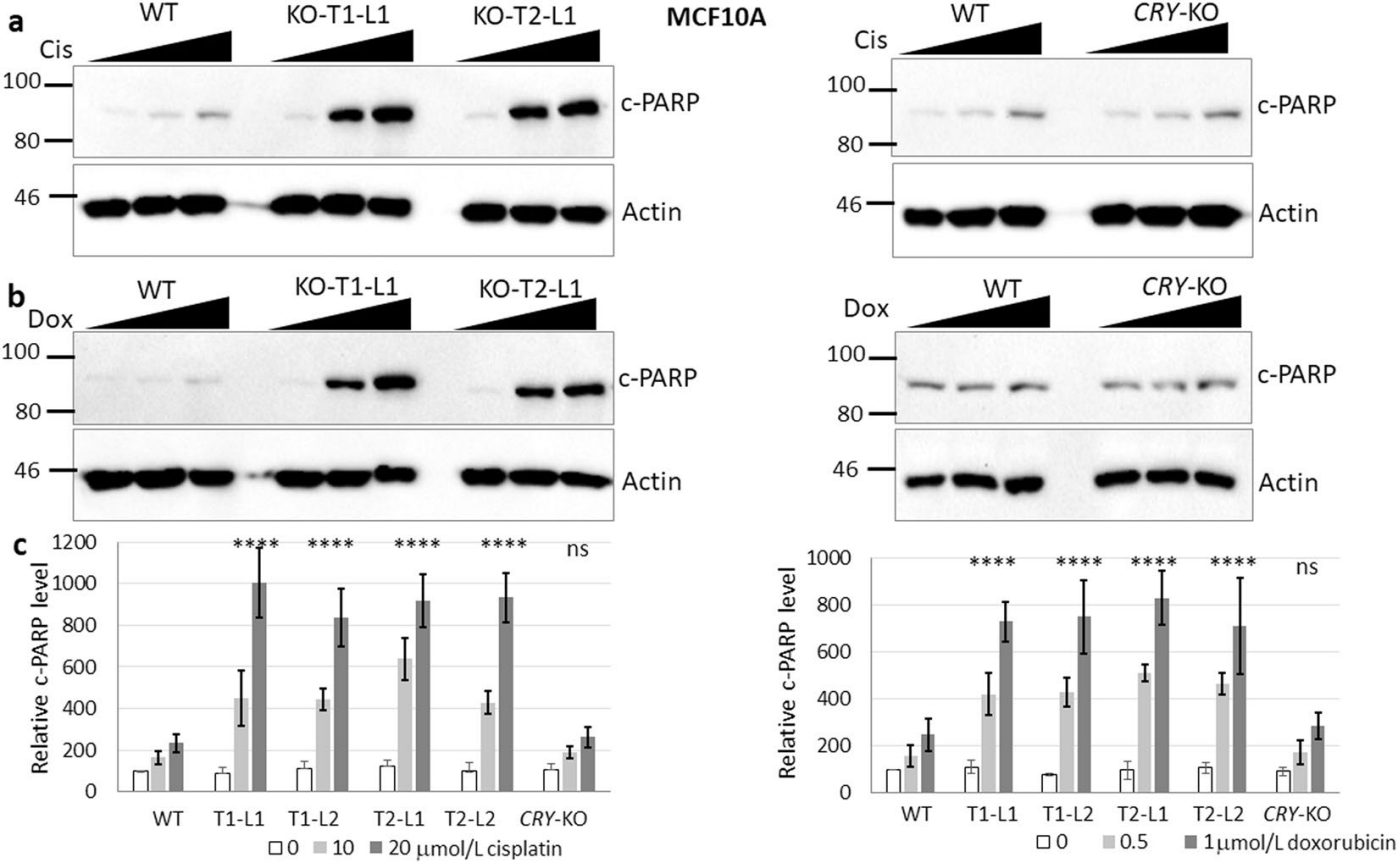
Effect of *BMAL1* mutations on apoptosis induced by cisplatin and doxorubicin agents in *p53* wild-type MCF10A cells. **(a**,**b)** Cells were treated with 0, 10, and 20 μmol/L cisplatin or 0, 0.5, and 1 μmol/L doxorubicin for 16 hours. Cell lysates were probed for cleaved PARP (c-PARP) by immunoblotting. Actin served as a loading control. (**c**) Quantifications of the c-PARP levels normalized to actin are plotted, which show that *BMAL1* mutations sensitized these cells to cisplatin- or doxorubicin-induced apoptosis. The data are the mean of three independent experiments ± SD. P-values are given for the apoptosis induced by 20 μmol/L cisplatin or 1 μmol/L doxorubicin in *BMAL1* knockouts relative to the parental cells. Numbers and marks on the left of each figure indicate the positions of the corresponding molecular size markers in kDa. KO indicates *BMAL1* knockouts generated by T1 or T2 sgRNA targeting, L indicates subclone for each sgRNA targeting. Relative c-PARP was analyzed by two-way ANOVA followed by Tukey’s post hoc tests for pairwise comparisons. ****p < 0.0001, ns; not significant (the details are available in Supplementary Table 1). Full-length blots are presented in Supplementary Fig. S9.

Our previous studies showed that the type of DNA-damage is important for the modulation of apoptosis by the circadian clock^16,35,36^. For example, mutation of *Cry* sensitizes *p53*-null cells to apoptosis when cells are treated with UV or UV-mimetics but not with double strand break-inducing agents such as doxorubicin^19,28^. To understand the effect of the disruption of the *BMAL1* gene on DNA damage-induced apoptosis, we analysed the effect of cisplatin, which causes bulky DNA-adducts. Dimers caused by this agent are repaired by nucleotide excision repair, or can initiate apoptosis if they cannot be repaired in time^35^. We conducted apoptosis assays at different doses and probed for apoptosis by measuring PARP cleavage^16^ over a period of 16 hours from the start of drug treatment. Doses were selected based on the linear response in wild-type MCF10A (Supplementary Fig. S2). The results are shown in Fig. 2 and Supplementary Fig. S3 (for L1s and L2s for each sgRNA, respectively). We found that *BMAL1* mutation sensitized *p53* wild-type MCF10A cells to cisplatin-induced apoptosis, and a very similar pattern was obtained with four different cell lines (Fig. 2a and Supplementary Fig. S3a). *CRY*-DKO cells served as a control, as these cells do not have cisplatin-induced apoptosis on a *p53* wild-type background^16,20,37^. Therefore, the sensitization effect observed in the presence of *BMAL1* knockout mutation is different from the *CRY* knockout mutation effect on apoptosis because *CRY* knockout mutation sensitizes cells to cisplatin-induced apoptosis only when *p53* is mutated, and the MCF10A cell line has a functional (wild-type) *p53*, which accumulates following DNA damage.

### The effect of *BMAL1* knockout mutation on apoptosis induced by the double strand break-inducing agent doxorubicin

Major lesions generated by UV-mimetics are repaired by the nucleotide excision repair pathway. On the other hand, doxorubicin induces double strand breaks, which are repaired by nonhomologous end-joining and homologous recombination^38^. Although previous studies showed that doxorubicin-induced apoptosis is not increased by a *CRY* mutation, the increase of cisplatin-induced apoptosis by *BMAL1* knockout mutation in the presence of wild-type *p53* (in MCF10A) prompted us to investigate doxorubicin-induced apoptosis. The results are shown in Fig. 2b and Supplementary Fig. S3b (for L1s and L2s for each sgRNA). *BMAL1* knockout mutation amplified doxorubicin-induced apoptosis as much as or at a comparable rate to that of cisplatin-induced apoptosis. Interestingly, it has been reported that *PER2* silencing effectively sensitizes MDA-MB-231 breast cancer cells to the cytotoxic effects of doxorubicin^39^. This suggests that different combinations of clock gene mutations and genotoxic agents may produce similar phenotypes.

### Enhancement of DNA damage-induced apoptosis by *BMAL1* knockout mutation

**Figure 3:**
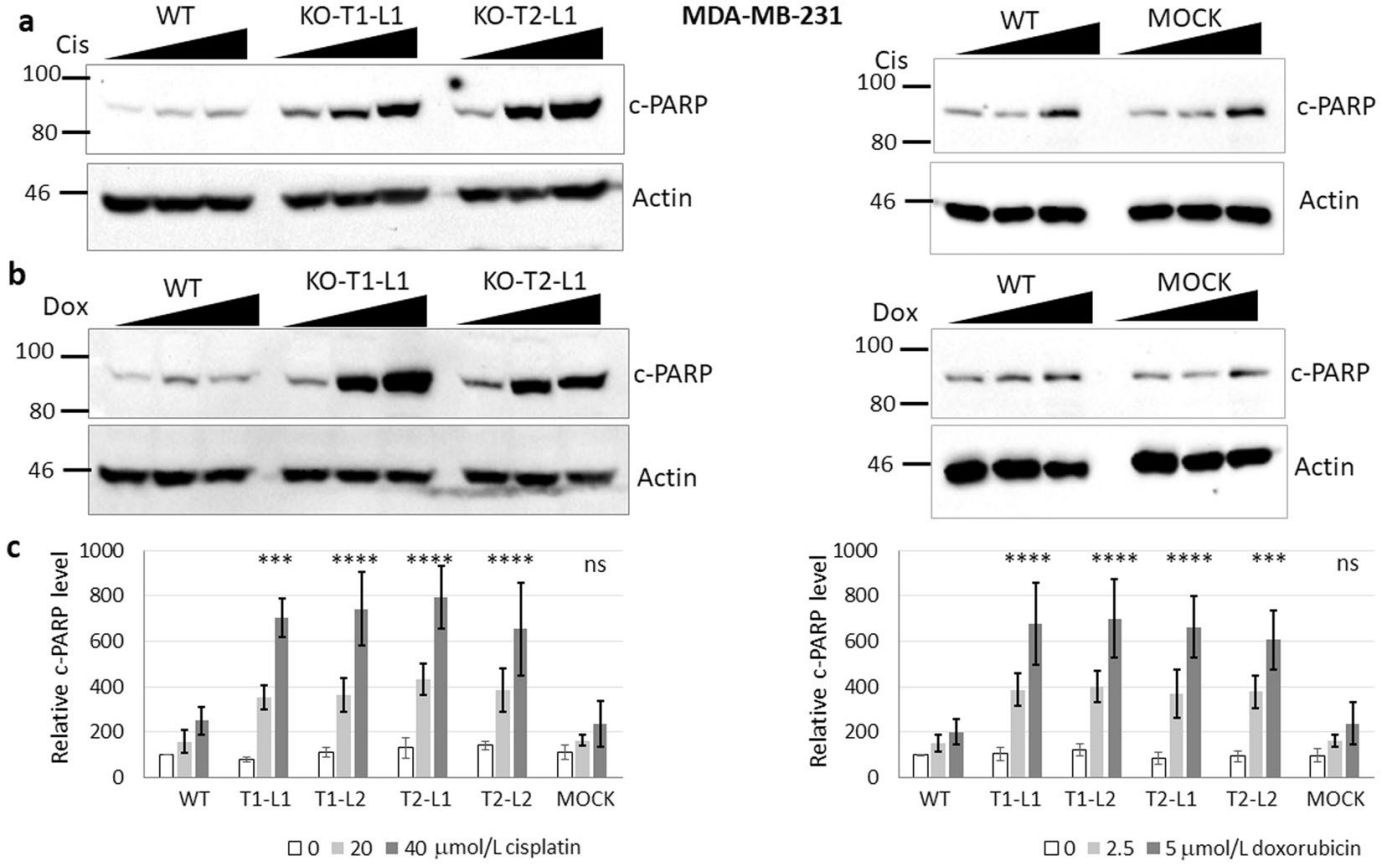
Effect of *BMAL1* mutations on apoptosis induced by cisplatin and doxorubicin agents in *p53* mutant cell line MDA-MB-231. (**a**,**b**) Cells were treated with 0, 20, and 40 μmol/L cisplatin or 0, 2.5, and 5 μmol/L doxorubicin for 16 h. Cell lysates were probed for cleaved PARP (c-PARP) by immunoblotting. Actin served as a loading control. (**c**) Quantifications of the c-PARP levels normalized to actin are plotted and show that *BMAL1* mutations sensitized these cells to cisplatin or doxorubicin-induced apoptosis. The data are the mean of three independent experiments ± SD. P-values are given for the apoptosis induced by 40 μmol/L cisplatin or 5 μmol/L doxorubicin in *BMAL1* knockouts relative to the parental cells. Numbers and marks on the left of each figure indicate the positions of the corresponding molecular size markers in kDa. KO indicates *BMAL1* knockouts generated by T1 or T2 sgRNA targeting, L indicates subclone for each sgRNA targeting. Relative c-PARP was analysed by two-way ANOVA followed by Tukey’s post hoc tests for pairwise comparisons. ***P < 0.001, ****p < 0.0001, ns; not significant (the details are available in Supplementary Table 1). Full-length blots are presented in Supplementary Fig. S10.

**Figure 4:**
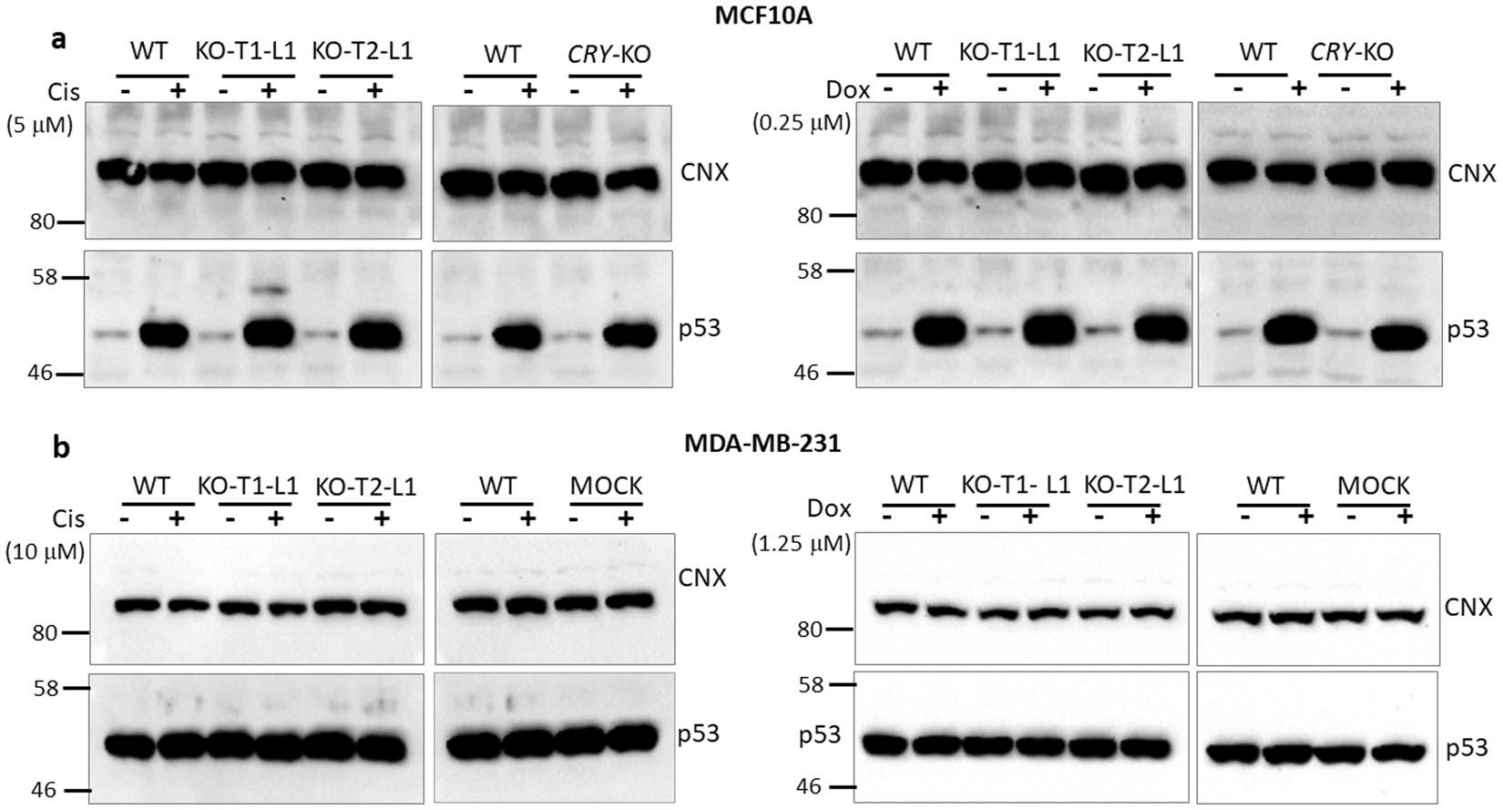
The effect of *BMAL1* knockout mutation on p53 protein accumulation following cisplatin or doxorubicin treatment. (**a**,**b**) MCF10A and MDA-MB-231 cells express wild-type and mutant p53 proteins, respectively. *BMAL1* mutation causes an increase in apoptosis in both cell lines while wild-type p53 accumulates at similar levels in wild-type and *BMAL1* (as well as *CRY*-DKO) knockouts in MCF10A clones. A similar increase in apoptosis by *BMAL1* mutations in untransformed (a) and transformed (b) suggests that this event is independent of the transformation status of the cells. A mock subclone (transduced with *BMAL1*-targeting lentivirus but with wild-type *BMAL1*) of MDA-MB-231 was used as a control instead of *CRY*-DKO, because *CRY*-DKO on a *p53* mutant background caused an increase in cisplatin-induced apoptosis in MDA-MB-231 cells, in agreement with previous reports (see Supplementary Data). Calnexin (CNX) served as a loading control. Numbers and marks on the left of each figure indicate the positions of the corresponding molecular size markers in kDa. KO indicates *BMAL1* knockouts generated by T1 or T2 sgRNA targeting, L indicates subclone for each sgRNA targeting. Full-length blots are presented in Supplementary Fig. S11.

We also speculated that the increase in apoptosis in the presence of *BMAL1* knockout mutation may be caused by a different mechanism than that of *Cry* knockout mutation in *p53* null cells, as reported previously^16^, because the sensitization to apoptosis by *BMAL1* gene knockout operates in the presence of wild-type *P53*, in opposite of the sensitization to apoptosis by *Cry* knockout only in the presence of mutant *p53*. We obtained similar results with MDA-MB-231 clones as those of MCF10A. *BMAL1* deletion amplified the both cisplatin and doxorubicin-induced apoptosis (Fig. 3 and Supplementary Fig. S4). The presence of a non-functional *p53* along with a *CRY* knockout mutation slightly enhanced cisplatin-induced apoptosis in our control *CRY*-DKO clone compared to the parental cell line (Supplementary Fig. S4), which confirms our previous studies^16,19^ that a *CRY* deletion sensitizes the cells cisplatin-induced apoptosis in the presence of *p53* mutation. We have confirmed the *CRY*-DKO status with immunoblotting using specific anti-CRY1 and anti-CRY2 antibodies (Supplementary Fig. S4). While treatment of MCF10A and its knockout subclones with both cisplatin and doxorubicin caused p53 accumulation at similar levels (Fig. 4a and Supplementary Fig. S5), as expected, the MDA-MB-231 parental line and its subclone cells had already an accumulation of p53 protein because of a mutation in the *p53* gene (Fig. 4b and Supplementary Fig. S5). Therefore, we used a mock control instead of *CRY*-DKO knockout MDA-MB-231 in addition to parental cell line for apoptosis assays. The mock control was a *BMAL1* targeting lentivirus-infected subclone, which did not knock out *BMAL1* (Fig. 1b). We could not detect any noticeable change in the population doubling times of the knockout cell lines compared to their parental cell lines (Supplementary Fig. S6).

### *BMAL1* knockout mutation increases the invasive capacity of transformed cells

**Figure 5:**
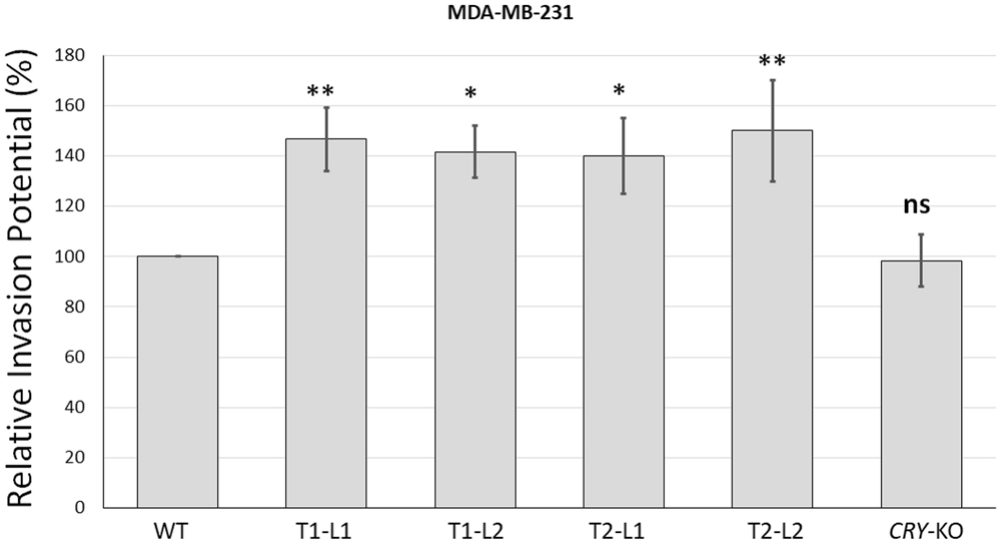
Effect of *BMAL1* mutations on MDA-MB-231 cell invasion. Matrigel invasion assay of MDA-MB-231 cells and its knockout subclones were performed using matrigel-coated invasion plates. Cells were seeded at 150,000 cells/well and allowed to invade toward 10% FBS for 36 hours. Invasive cells on the bottom of the invasion membrane were stained (and quickly checked under a microscope) and quantified at OD 560 nm after extraction. There was a similar number of cells in the parent and knockout cell samples. The results of invasion are presented as means ± SD of three independent experiments; SD is denoted by error bars. Invasion was analysed by one-way ANOVA followed by Tukey’s post hoc tests for pairwise comparisons. *P < 0.05, **P < 0.01, ns; Not significant. KO indicates BMAL1 knockouts generated by T1 or T2 sgRNA targeting, L indicates subclone for each sgRNA targeting.

Overall, the amplification of apoptosis by *BMAL1* knockout mutation in MCF10A could suggest that *BMAL1* deletion might have an anti-tumor effect in certain types of cells. However, induction of apoptosis before transformation or during eradication of cancer cells for therapeutic purposes should be considered in overall effect of *BMAL1* mutations. Another pathway which is important for therapeutic intervention of cancer spread is invasion potential of cancer cells. Therefore, we analysed the effect of *BMAL1* deletion on the invasion capacity of MDA-MB-231 cells which might be more important process than apoptosis in malignancy. Tumor cells need to break the basement membrane to confer a malignant phenotype or to form (micro) metastases. During this process, cells obtain invasive properties which can be measured *in vitro*. MDA-MB-231 cells are an invasive breast cancer cell line, and therefore we used these cells instead of non-tumorigenic MCF10A cells for an *in vitro* invasion assay. *BMAL1* knockout mutation increased the invasion into the basement membrane compared to parental MDA-MB-231 cells (Fig. 5). Moreover, we used another control to eliminate any off-target effects induced by CRISPR by testing *CRY* mutant MDA-MB-231 cells in parallel.

## Discussion

It has been reported that *Cry* mutation renders the Ras-transformed *p53*-null cells, but not cells with wild-type *p53*, more susceptible to killing by agents that activate either the intrinsic or the extrinsic apoptosis pathways^20,37^. It has also been shown that the deletion of other clock genes does not affect apoptosis following treatment with a variety of DNA damaging agents including UV or UV-mimetics and double strand break-forming doxorubicin in fibroblasts isolated from different clock gene knockout mice^28^. Moreover, spontaneous and/or ionizing radiation-induced tumorigenesis has been performed using *Cry*, *Clock*, *Per or Bmal1* knockout mice in different laboratories. For *Per*, it has been reported that *Per2* deficiency increased irradiation-induced tumor incidence^13^, although another group did not obtain increased tumor incidence with *Per2* (or *Per1*) knockout^14^. In another study, it was shown that loss of *Per2* function accelerated lung cancer formation in mutant mice^24^. These findings with *Per2* and carcinogenesis suggest that the effect of *Per2* mutation in tumorigenesis should be further studied. Even though in early studies, *Bmal1* knockout mice were not a good model for studying cancer because these animals age early, following the accumulation of reactive oxygen species in tissues^23^, recently an inducible *Bmal1* knockout mouse model, which expresses the gene during embryogenesis but not in after birth, was generated to eliminate early aging phenotype^27^. *Clock* knockout mice did not have an increased incidence of cancer^22,23^ while whole-body knockout of *Bmal1* in mouse was recently associated with increased accelerated lung cancer^24^. In a similar organ specific targeting approach, Kettner *et al*. reported that hepatocyte-specific loss of *Bmal1* enhances the formation of spontaneous hepatocellular carcinoma^40^. On the other hand, a study by Puram *et al*. suggested that genetic deletion of *Bmal1* in established leukemias results in a competitive disadvantage and suppressed leukemogenesis^41^. In respect to the effect of *Cry* on carcinogenesis, it was reported that *Cry* DKO mice were not distinguishable from wild-type ones^15^, while *Cry2* deletion increased the cancer risk^21^. In opposite to *Cry* DKO, the deletion of *Cry* genes in *p53* null background had a protective effect against the spontaneous development of lymphomas^16^. Considering the findings with different outputs from animal models, we wanted to test the effect of circadian clock genes in specific cancer-related events in order to form a platform where we can add more factors quickly and study molecular events which might difficult or time-consuming at whole-organism level. We decided to knockout *Bmal1* gene in our model because the deletion of *Bmal1* can completely disrupt the circadian clock in opposite to the requirement of deletion of two genes for *Cry* or *Per* genes to disrupt the circadian clock completely. We tested carcinogenic events (such as apoptosis and invasion) using cell lines as models following knockout of *BMAL1* with CRISPR. Keeping in mind findings from previous studies which used fibroblasts, we thought that epithelial cells would be a better starting material because most tumors originate from these cells. As a result, the use of appropriate cell lines enabled us to test events such as apoptosis, which has a protective effect against cancer formation by eliminating abnormally behaving but still non-transformed cells, and invasion capacity in transformed cells, which has a cancer promoting effect by increasing metastasis or micro-metastasis. Epidemiological data suggest that disruption of the circadian clock in night-shift nurses or flight attendants increases breast cancer risk two fold^42^, suggesting that breast epithelial cell lines are an appropriate cell model. MCF10A is a nearly normal (nontumorigenic) cell line with wild-type *p53* and a resettable circadian clock in culture. Therefore, we studied its apoptotic response after treating parental cells and *BMAL1* knockout clones with bulky DNA adduct forming or double strand break forming agents, cisplatin and doxorubicin, respectively. First, *BMAL1* knockout sensitized the cells to cisplatin-induced apoptosis. We obtained similar results by targeting the *BMAL1* gene with two different sgRNAs. A *CRY*-null knockout (*CRY*-DKO) cell line was also used as a control to eliminate the possibility that lentiviral infection might sensitize cells to apoptosis. We found that *CRY*-DKO did not have an altered apoptotic response to cisplatin or doxorubicin in MCF10A cell line which expresses wildtype *p53*. It was reported that *CRY1* and *CRY2* play different roles in tumorigenesis^21^, therefore we preferred to use double-knockout (DKO) of *CRY1* and *CRY2* as the control because our experience with *CRY*-DKO cell lines showed that DNA-damage induced apoptosis is not altered in *p53* wildtype background. The increase in apoptosis by *BMAL1* knockout in MCF10A cells did not require a *p53* null background. This was different from the amplification of apoptosis by *Cry* mutation which can be observed only in *p53* mutant background^16^. More strikingly, we also observed strong amplification of the apoptotic response by *BMAL1* knockout following double strand break induction with doxorubicin. This increase in both UV-mimetic and double strand break damage-induced apoptosis in the same cell line was not observed with any other circadian clock protein mutations. Our finding that *BMAL1* deletion increases sensitivity to chemotherapeutic agents suggests that interfering with *BMAL1* expression might have a protective or preventive function against tumor initiation following repeated DNA damage insults. It has been reported that *BMAL1* alters cell growth and can control *p53* activation^43^. We did not observe a significant change in population doubling times in our knockouts compared to those of parental or control clones (Supplementary Fig. S6). More importantly, we concentrated on DNA damage-induced apoptosis and detected a significant sensitization in breast epithelial cells. It has also been reported that *BMAL1* overexpression sensitized tongue squamous cell carcinoma cells to apoptosis induced by an antineoplastic agent, paclitaxel^44^. However, DNA damage-induced and paclitaxel-induced apoptosis are different events, and this data does not conflict with our results. It has also been reported that *BMAL1* overexpression in colon cancer cell lines sensitized these cells to oxaliplatin^45^. Alternatively, *BMAL1* knockdown was found to suppress proliferation, and anchorage-dependent and independent clonal growth of malignant pleural mesothelioma cells^46^. Differentially from our work, it was shown that knockdown of *Bmal1* in murine colon cancer cells (C26) and fibroblast cells (L929) decreased apoptosis induced by etoposid (VP16) and decreased DNA damage induced by cisplatin^47^. One possibility is that low level of Bmal1 protein in shRNA treated cells might be enough to sustain a functional circadian clock while complete knockout of *BMAL1* gene disrupt the circadian clock. This difference between the full blown disruption or attenuation the circadian clock may change the effect of *BMAL1* on the sensitivity to double-strand break or bulky-adduct forming drugs. It is also possible that the modulation of cells to DNA damages by *BMAL1* gene can be cell or tissue-specific. These reports with different findings suggest that overexpression in the presence of endogenous *BMAL1* and depletion of *BMAL1* through small interfering RNA or genome editing may result in different outputs depending on the origin of the cells and type of chemotherapeutic drugs.

Then, we checked if *BMAL1* mutation had a protective effect by decreasing the invasion of transformed cells. In fact, it has been reported that the knockdown of *BMAL1* by RNA interference promoted cancer cell invasion independent of *p53* status in lung cancer and glioma cells^48^. On the other hand, it has been reported that *Bmal1* inhibits invasion in tongue squamous cell carcinoma cells and telomerase reverse transcriptase gene (*hTERT*) mediates Bmal1-driven sensitivity to an antineoplastic agent paclitaxel^44^. *BMAL1* also promoted migration and invasion in an immortalized human extravillous trophoblast cell line HTR-8/SVneo^49^. In the present study, we used an invasive breast cancer cell line, MDA-MB-231, to analyse the effect of *BMAL1* mutation on invasion. *BMAL1* knockout mutation increased invasive capacity more than ~40%, which was statistically significant. Additionally, for the invasion assay, we obtained similar results from *BMAL1* knockout cells which were generated by targeting the gene different sgRNAs. We used the parental cell line and a *CRY*-DKO of the MDA-MB-231 cell line as a reference. *CRY* knockout mutation did not change invasive capacity significantly under the same conditions. This might suggest the effect of *BMAL1* on the invasion might be circadian clock-independent because deletion of *CRY* genes did not have the same phenotype. On the other hand, these effects may be cell type specific even though we used one transformed and one untransformed cell line to reduce this risk. This cell type specific phenotype might be the result of a combination of circadian-dependent and -independent functions with the genetic make-up of the cell lines. Actually, this is also true for the tissue specific effects in whole-body knockouts depending on the circadian-dependent and -independent functions of *Bmal1* genes in different organs. For example, in *Bmal1* knockouts, accumulations of reactive oxygen species are not the same in all cell types^23^. Keeping in mind that platforms or models with cell lines have technical difficulties to reflect *in vivo* events, it may offer a very easy modifiable, cheaper, and much quicker working material to study molecular events. For example, testing the relationship between *Bmal1* and another gene for example with *p53* would be much easier just by knocking out *p53* in *Bmal1* null cells.

**Figure 6:**
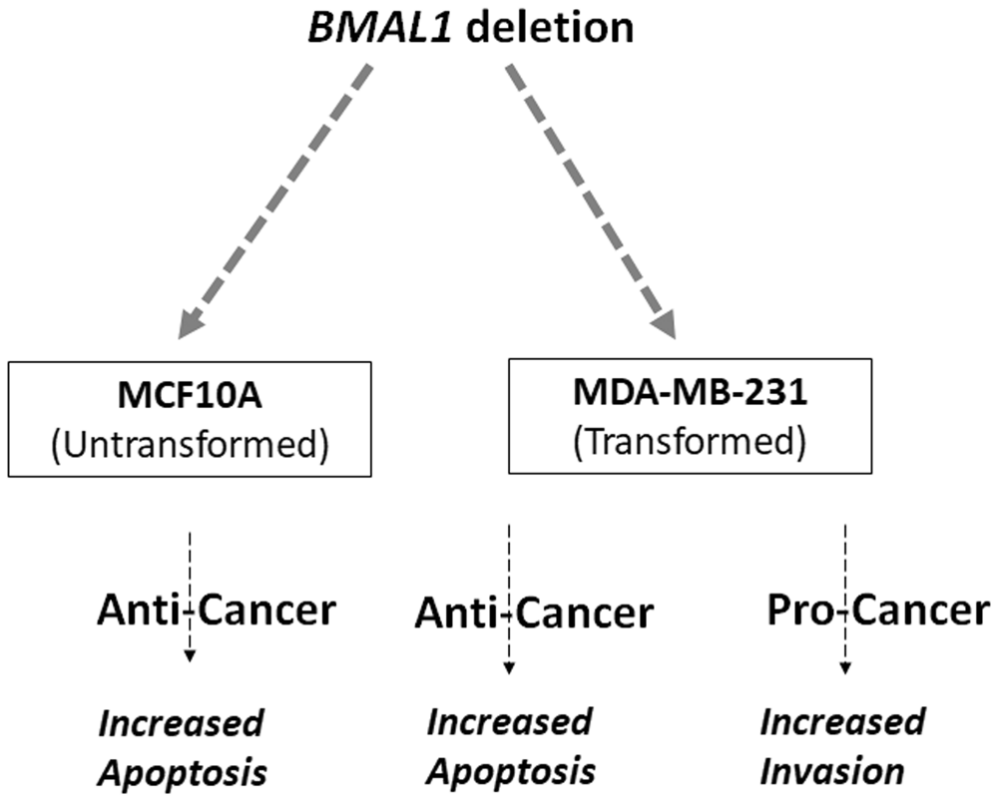
Pro- and anti-cancer effects of *BMAL1* knockout mutation. Knockout of *BMAL1* by CRISPR in non-tumorigenic MCF10A and invasive MDA-MB-31 cell lines had an anti-cancer effect by sensitizing these cells to the genotoxic agents cisplatin and doxorubicin. On the other hand, *BMAL1* knockout in MDA-MB-231 cells increased their invasive potential, which reflects a pro-cancer effect. Even though *BMAL1* knockout increased the invasive potential in MDA-MB-231 cells, it increased apoptosis in the same cells. This suggests that the circadian clock gene *BMAL1* has both anti- and pro-cancer effects in certain context.

Altogether, there are different findings from different laboratories in respect to the relationship between cancer and circadian clock as discussed above. Based on our finding, we observed that the same knockout mutation had both protective and promoting functions in two breast epithelial cell lines (Fig. 6).

We have selected BMAL1 gene for knocking out for the following reason: *BMAL1* is the only gene whose deletion alone leads to complete loss of rhythmicity. Other than *BMAL1* gene, the disruption of the circadian rhythm requires knockouts of at least two genes such as *CRY1* and *CRY2* or *PER1* and *PER2*, which would increase off-targets by two different sgRNAs. However, it is also known that *BMAL1* has also non-circadian functions. For example, germline *Bmal1* knockout is associated with early aging while inducible knockout mouse does not have such gross effects while both are deficient in the circadian clock^27^. Moreover, *Cry1 & Cry2* or *Per1* &*Per2* double knockouts do not have aging properties or some other metabolic abnormalities observed in *Bmal1* knockout mice^50^. In fact, the increase in the invasion properties in *BMAL1* knockout MDA-MB-231 cells was not observable in *CRY* DKO MDA-MB231 cells (Fig. 5), this suggests that this might operate through non-circadian functions of *BMAL1* gene. In opposite view, using two sgRNAs to knockout *CRY1* and *CRY2* might have induced an unforeseen off-target effect which might have cancelled the increased invasions properties. In any case, the deletion of *BMAL1* gene sensitized an untransformed and a transformed breast epithelial cell line to cisplatin or doxorubicin-induced apoptosis while it enhanced invasion capacity of transformed MDA-MB-231 cell line either a circadian-dependent or -independent way.

In summary, we found that knockout of *BMAL1* gene in two mammalian cells affects the apoptotic response and invasion properties differently in respect to the contribution to carcinogenesis. Cell-based *in vitro* circadian systems have been regarded as poor models to study the circadian clock’s effect on carcinogenesis. However, this study suggests that appropriate modelling of events *in vitro* might reveal new findings which were not detectable in animal models. On the other hand, we wish to emphasize that even though cell-based systems such as in this work are useful for studying molecular events of basic circadian clock, they have limitations to reflect circadian clock-associated phenotypes observed *in vivo*. Therefore, our findings should be interpreted from the view that circadian clock gene *BMAL1* affects the molecular events associated with carcinogenesis differentially but these findings do not validate or invalidate the findings from *in vivo* animal models because there are more factors than what we investigated.

## Methods

### Cell culture

MCF10A, MDA-MB-231, and HEK293T cell lines were from American Type Culture Collection (ATCC; Rockville, MD), and maintained in a humidified incubator at 37 °C under 5% CO_2_. MCF10A, a nontumorigenic and nearly normal breast epithelial cell line, was initially grown in Mammary Epithelial Basal Medium (MEBM; Lonza, Walkersville, MD, USA) supplemented with SingleQuots (MEGM BulletKit; Lonza) and 100 ng/mL cholera toxin (Lonza) for initial growing and stocking purposes. For experimental procedures such as lentiviral infection and all remaining assays, MCF10A was grown and cultured in a Dulbecco’s Modified Eagle Medium (DMEM)-based medium (Thermo Fisher Scientific, Waltham, MA, USA), containing 20 ng/mL epidermal growth factor (Sigma, St. Louis, MO, USA), 0.5 mg/mL hydrocortisone (Sigma), 10 μg/mL insulin (Sigma), 1% nonessential amino acids (Thermo Fisher Scientific), 100 U/mL penicillin, 100 μg/mL streptomycin (Thermo Fisher Scientific), and 10% fetal bovine serum (FBS; Thermo Fisher Scientific). All transfections were done using early passages (<p20) of MCF10A, and p53 status was occasionally checked by treating the cells with 5 μM cisplatin and analysing DNA damage-induced p53 protein accumulation with immunoblotting. MDA-MB-231 and HEK293T cell lines were grown and maintained in DMEM supplemented with 10% FBS, 100 U/mL penicillin, 100 μg/mL streptomycin, and 1% non-essential amino acids (all from Thermo Fisher Scientific). The HEK293T cell line was used to generate lentivirus particles. MCF10A and MDA-MB-231 were validated by short-tandem repeat (STR) analyses, and the STR profiles were cross-checked against the ATCC database. Both cell lines had ≥80% match with the ATCC online STR database and were thus considered valid^51^. All parental and subclone lines were tested for mycoplasma contamination using the e-Myco plus Mycoplasma PCR Detection Kit (Boca Scientific Inc., Boca Raton, FL, USA) (see Supplementary Fig. S7).

### Establishment of knockout cell lines

MCF10A and MDA-MB-231 cells were genome-edited with CRISPR^31^ using the CRISPR Design Tool^29^ single-guide RNA (sgRNA) design program to select the most efficient sgRNA for the target genes. Double stranded oligos (of which sequences are given below and were purchased from Invitrogen, Thermo Fisher Scientific) were annealed in microcentrifuge tubes containing in 1× NEB buffer 2 solution (New England Biolabs, Hertfordshire, U.K.) by heating to 95 °C on a heat block for 5 min, then cooling to ambient temperature. Annealed oligos (100 ng) were ligated into 50 ng of LentiCRISPRv2^52^ digested with BsmBI. Ligation products were transformed into ultracompetent Stbl3 *Escherichia coli* cells. Half of the ligation products were mixed with 100 μL competent cells, and then kept on ice for 30 min. The cells were exposed to a heat shock at 42 °C for 60 s and transferred back into ice for 2 min. Approximately 900 μL of Luria–Bertani (LB) medium was added to the cells, which were then incubated for 1 h at 37 °C with rotating. To select single colonies, 100 μL of the transformation products was plated on LB-Agar plates containing 100 μg/mL ampicillin. Selected colonies were confirmed by DNA sequencing using the primer U6-F (5′-GAGGGCCTATTTCCCATGATT-3′).

LentiCRISPRv2-based CRISPR constructs were co-transfected with pCMV-dR8.2 dvpr and pCMV-VSVG packing plasmids into HEK293T cells to produce lentiviral particles as previously described^53^. Media containing lentiviral particles were collected following 48 h of transfection, filtered with a 0.2-μm filter, and kept at −80 °C. MCF10A and MDA-MB-231 cells were infected with lentiviral particles in 12-well plates in 1 mL of total medium (0.5 mL fresh culture medium and 0.5 mL medium containing viral particles) containing 8 ng/mL polybrene (Santa Cruz, TX, USA). Infected cells were selected with puromycin (0.5 mg/mL for MCF10A and 1.5 mg/mL for MDA-MB-231) for 3–4 days, and then single cell-derived clones were grown and picked by serial dilution. Candidates were grown in six-well plates in duplicate, and one was used for immunoblotting analysis to select candidate knockouts for further confirmation. The *BMAL1* gene was targeted at two genomic locations separately to reduce nonspecific off-target effects of random integration or selection. Two single cell-derived cell lines were chosen to be used in further experiments for each targeting sgRNA. The guide sequences targeting the human *BMAL1*, *CRY1*, and *CRY2* genes are as follows:

*BMAL1* T1 Sense: 5′CACCGTGTTCTGTATATTCTAACCT 3′;

Antisense: 5′ AAACAGGTTAGAATATACAGAACAC 3′;

*BMAL1* T2 Sense: 5′CACCGTAGATAAACTTACTGTGCTA 3′;

Antisense: 5′ AAACTAGCACAGTAAGTTTATCTAC 3′;

*CRY1* Sense: 5′ CACCGCCTTCAGGGCGGGGTTGTCG 3′;

Antisense: 5′ AAACCGACAACCCCGCCCTGAAGGC 3′;

*CRY2* Sense: 5′CACCGCTGCGACTCCACGACAACC 3′;

Antisense: 5′ AAACGGTTGTCGTGGAGTCGCAGC 3′.

Mutations caused by CRISPR-Cas9 T1 and T2 sgRNAs were described by T7 Endonuclease assay (see supplementary data for details) and Sanger sequencing from PCR products as described previously^33^.

### Western blotting

The cells were harvested and lysed in radioimmunoprecipitation assay (RIPA) buffer (50 mmol/L Tris-HCl pH 7.5, 150 mmol/L NaCl, 1% IGEPAL, 0.5% deoxycholate, and 5 mmol/L EDTA) containing a protease inhibitor cocktail (Sigma). For protein immunoblot analysis, 50 μg of the cell lysate was resolved using Sodium Dodecyl Sulphate-Polyacrylamide Gel Electrophoresis and transferred onto nitrocellulose membranes (Bio-Rad, Richmond, CA,. USA). The membranes were blocked in a solution of 1× Tris-Buffered Saline and Tween (TBST; 150 mmol/L NaCl, 20 mmol/L Tris-HCl, pH 7.4, and 0.1% Tween 20) containing 5% non-fat dry milk, and then incubated with primary antibodies in the blocking solution overnight at 4 °C. The membranes were washed 4 times with 1× TBST at room temperature to remove unbound antibodies, and incubated with horseradish peroxidase (HRP)-conjugated secondary antibodies in the blocking solution for 1 h on an orbital shaker. Unbound antibodies were again washed away four times with 1× TBST, and chemiluminescence was developed using SuperSignal West Femto Maximum Sensitivity Substrate (Thermo Fisher Scientific). Images were captured using the ChemiDoc XRS+ system (Bio-Rad). The following antibodies were used to detect their respective proteins: Bmal1, Cryptochrome 1, Cryptochrome 2 (Bethyl Labs Inc. Montgomery, TX., USA); p53, cleaved PARP, actin (Cell Signaling Technology, Boston, MA, USA); Calnexin, HA tag (Santa Cruz). HRP-labeled anti-mouse and anti-rabbit antibodies (Cell Signaling Technology) were used at 1:5000 dilution.

### Apoptosis assay

Apoptosis was induced by treating cells with a bulky-DNA adduct forming agent, cisplatin, or a double strand break forming agent, doxorubicin (Sigma). Cisplatin (Sigma) stock at 5 mM was dissolved in 0.9% (w/v) NaCl solution. Doxorubicin was prepared at 5 mM in double distilled sterile water. The cells were treated with cisplatin or doxorubicin at doses from 0 to 40 μM and 0 to 5 μM, respectively, in cell culture media for 16 hours. Apoptosis was assayed by detecting cleaved PARP, and was normalized to actin.

### Invasion assay

Invasion assays were performed using the CytoSelect 24-Well Cell Migration Assay kit (Cell Biolabs, San Diego, CA, USA) following the manufacturer’s instructions. Briefly, basement membranes (coated on a polycarbonate filter with an 8-μm pore size) were reconstituted with 300 μL of serum-free DMEM medium for 1 h at room temperature. MDA-MB-231 and its knockout clones were trypsinized and washed once with DMEM containing 10% FBS, and then were washed twice with serum free-DMEM. The cells were resuspended in DMEM containing 0.1% Bovine Serum Albumin (Sigma) at a density of 5 × 10^5^ cells/mL. The cell suspensions (300 μL) were seeded into the upper chambers, and 500 μL of DMEM medium containing 10% FBS was added to the lower chambers. The cells were allowed to invade for 36 h in a CO_2_ incubator, fixed, stained, and quantitated by measuring absorption at 560 nm using a Synergy H1 microplate reader (BioTek Instruments, Inc., Winooski, VT, USA,). Invasion was also checked under a microscope by focusing to the lower part of the Matrigel to control the invasion potential of the cell lines. Invasive potential was normalized to that of wild-type MDA-MB-231 parental cells.

### Statistical analysis

Values are shown as mean ± SD of at least three experiments. Results were statistically analysed by (two-way or one-way) ANOVA followed by Tukey’s post hoc tests for pairwise comparisons using GraphPad Prism 7.04 (GraphPad Software, Inc., San Diego, USA). Types of the tests for figures were indicated in the figure legends.

## Electronic supplementary material

Supplementary Information

## Data Availability

All data generated or analysed during this study are included in this published article and its Supplementary Information file.
