# Supplementary material for: Opposite Carcinogenic Effects of Circadian Clock Gene BMAL1
Source: Sci Rep. 2018 Oct 30;8:16023. doi: 10.1038/s41598-018-34433-4 (PMC6207783; doi:10.1038/s41598-018-34433-4)

Opposite Carcinogenic Effects of Circadian Clock Gene *BMAL1*

Tuba Korkmaz, Fatih Aygenli, Handan Emisoglu, Gozde Ozcelik, Asena Canturk, Secil Yilmaz, Nuri Ozturk*

*Corresponding Author: nuriozturk@gtu.edu.tr

Department of Molecular Biology and Genetics, Gebze Technical University, Gebze, Kocaeli, Turkey

Supplementary Data

Supplementary Text

Supplementary Methods

T7 Endonuclease Assay

Genomic DNA was extracted from approximately 10^6 cells using the NucleoSpin kit (Macherey-Nagel, GmbH & Co. KG, Germany) following the manufacturer's instructions and the genomic sequences spanning the target sequences of sgRNA's were PCR-amplified by Taq DNA Polymerase (New England Biolabs, Hertfordshire, U.K.) using specific forward and reverse primers, of which sequences are given below. The PCR conditions were a denaturation step at 95^oC for 5 minutes; then 35 cycles at 95 ^oC for 30 seconds, 60 ^oC for 20 seconds, and 68 ^oC for 30 seconds; and a 2-minute final extension at 68 ^oC. PCR products were column purified using NucleoSpin Gel and PCR Clean-up kit (Macherey-Nagel). 200 ng from wild-type and mutated cleaned PCR samples were denatured and reannealed for heteroduplex formation in 1x NEB buffer 2 (New England Biolabs) by heating to 95 ^oC for minutes and allowing the samples to cool down after turning the heat block off. Finally, 10 µl of heteroduplex formation reaction product was incubated with 2.5 unit of T7 Endonuclease (New England Biolabs) at 37°C for 2 hours, and the digested products were analyzed with the agarose gel electrophoresis.

Sequences of right and left primers for T7Endonuclease assay are shown below.
BMAL1-T1_F and R: 5' GACAAAGATGACCCTCATGGA 3' and 5' AGCCAGTATGGAGAGCATGG 3'
BMAL1-T2_F and R: 5' CCTGGCAGTGAGACCATTTT 3'and 5' TCACTTCATTTGGCATCACG 3'

Confirmation of Knockouts by Sanger Sequencing

In order to confirm CRISPR-Cas9 mediated disruption of coding sequences in our *BMAL1* knockouts, we amplified full-length *BMAL1* cDNAs from wildtype and their T1 and T2 sgRNA targeted knockouts of both MCF10A and MDA-MB-231. For this purpose, we converted total RNA into cDNA using ProtoScript II reverse transcriptase and oligo-dT by following the manufacturer's protocol (New England Biolabs). Full-length cDNAs (which encompasses the expected Cas9 cut sites at ~180 bp and ~342 bp following the first base of start codon for T1 and T2 respectively)

were amplified using the primers with NheI and EcoRI sites to aid directional cloning. Forward and reverse oligo sequences used to amplify full-length cDNAs from our cell lines are shown below.

NheI-BMAL_F 5’ CAAGCTAGCGCCACCATGGCAGACCAGAGAATGGACA 3’

EcoRI-BMAL_R 5’ AAAGAATTCCAGCGGCCATGGCAAGTCACT 3’

For T1 mutations, PCR products from cDNAs of knockout or wildtype cells were sequenced using NheI-BMAL1_F oligo. Because we could not get clear sequencing for T2 sites using NheI-BMAL1_F oligo, we sequenced T2 mutation from gDNA after amplifying with T7 Endonuclease oligos (of which sequences were given in T7 Endonuclease Assay section). PCR products were sequenced using the BMAL1-T2_R oligo. Alignments of mutations read by Sanger sequencing were done using an online tool (CRISP-ID: Detecting CRISPR mediated indels by Sanger sequencing tool)[1]. This tool was used from an online service at http://crispid.gbiomed.kuleuven.be/.

Population Doubling (PD) assay

For the population doubling (PD) assay, $50 x 10^3$ cells were seeded onto 6-well plates, and counted manually using a hemocytometer after 48 hours. PD was calculated by the formula: PD=Log (Nf/Ni)/Log2, where Nf the number of cells counted and Ni the number of cells seeded.

Analysis of mycoplasma status of cell lines

The contamination with mycoplasma in cells was tested using polymerase chain reaction (PCR) based e-Myco Plus Mycoplasma PCR Detection Kit (Boca Scientific Inc., Boca Raton, FL, USA) according to the procedure recommended by the manufacturer. Shortly, genomic DNAs (50 ng) of cell lines, which were kept in the same culture medium for 5 days, were added to the tubes containing the reconstituted PCR mix. Primers were included in the mix for a housekeeping gene and for common species of mycoplasma.

Reference:

1 Dehairs, J., Talebi, A., Cherifi, Y. & Swinnen, J. V. CRISP-ID: decoding CRISPR mediated indels by Sanger sequencing. *Sci Rep* **6**, 28973, doi:10.1038/srep28973 (2016).

Supplementary Table

Supplementary Table 1. The detail of the statistical methods used for the analyses of apoptosis and invasion assays.

a- Statistical Analyses for Apoptosis Assays

**Two-way ANOVA Table for Variance Analysis**						
**Source of Variation**	**% of Total Variation**	**SS**	**DF**	**MS**	**F value**	**P value**
MCF10A Cells: 0µM-10µM-20µM Cisplatin						
Interaction	17.18	990565	10	99057	14.56	<0.0001****
Between Cells	55.09	3177281	2	1588641	233.6	<0.0001****
Doses of Drug	23.49	1354536	5	270907	39.83	<0.0001****
Residual		244866	36	6802		
MCF10A Cells: 0µM-0.5µM-1.0µM Doxorubicin						
Interaction	13.18	498907	10	49891	7.572	<0.0001****
Between Cells	58.45	2212493	2	1106246	167.9	<0.0001****
Doses of Drug	22.1	836654	5	167331	25.4	<0.0001****
Residual		237200	36	6589		
MDA-MB-231 Cells: 0µM-20µM-40µM Cisplatin						
Interaction	14.15	465530	10	46553	6.122	<0.0001****
Between Cells	56.09	1845848	2	922924	121.4	<0.0001****
Doses of Drug	21.45	705965	5	141193	18.57	<0.0001****
Residual		273733	36	7604		
MDA-MB-231 Cells: 0µM-2.5µM-5.0µM Doxorubicin						
Interaction	13.98	397807	10	39781	5.024	0.0001***
Between Cells	54.32	1545881	2	772941	97.61	<0.0001****
Doses of Drug	21.68	617009	5	123402	15.58	<0.0001****
Residual		285067	36	7919		

**P (Tukey's post hoc tests for pairwise comparisons) MCF10A**					
**Drugs**	**WT vs. T1-L1**	**WT vs. T1-L2**	**WT vs. T2-L1**	**WT vs. T2-L2**	**WT vs. *CRY* DKO**
0µM-10µM-20µM Cisplatin	****	****	****	****	Ns
0µM-0.5µM-1.0µM Doxorubicin	****	****	****	****	Ns

**P (Tukey's post hoc tests for pairwise comparisons) MDA-MB-231**					
**Drugs**	**WT vs. T1-L1**	**WT vs. T1-L2**	**WT vs. T2-L1**	**WT vs. T2-L2**	**WT vs. Mock**
0µM-20µM-40µM Cisplatin	***	****	****	****	Ns
0µM-2.5µM-5.0µM Doxorubicin	****	****	****	***	Ns

Relative c-PARP signal was analyzed by two-way ANOVA followed by Tukey's post hoc tests for pairwise comparisons using GraphPad Prism 7.04 (GraphPad Software, Inc., San Diego, USA). The table shows results of comparisons between means of c-PARP level in wild-type and knockout cells exposed to different doses of cisplatin or doxorubicin. *P<0.05, **P<0.01 ***P<0.001, ****P<0.001, Ns: Not significant

b- Statistical Analysis for Invasion Assay

**One-way ANOVA Table for Variance Analysis**					
**Source of Variation**	**SS**	**DF**	**MS**	**F value**	**P value**
**MDA-MB-231 Cells**					
Between Cells	8444	5	1689	10.13	0.0006***
Residual	2000	12	166.7		
Total	10444	17			

**P (Tukey's post hoc tests for pairwise comparisons)**					
	**WT vs. T1-L1**	**WT vs. T1-L2**	**WT vs. T2-L1**	**WT vs. T2-L2**	**WT vs. *CRY* DKO**
**Invasion**	**	*	*	**	Ns

Invasion was analyzed by one-way ANOVA followed by Tukey's post hoc tests for pairwise comparisons using GraphPad Prism 7.04 (GraphPad Software, Inc., San Diego, USA). The table shows results of comparisons between means of invasion level in wild-type and knockout cells. *P<0.05, **P<0.01 ***P<0.001, ****P<0.001, Ns: Not significant

Supplementary Figure Legends

Figure S1. Confirmation of CRISPR-Cas9 mediated mutations. T7 endonuclease assay was used to confirm genome editing from genomic DNA (**a**). The genomic DNA from wildtype and mutated samples were PCR-amplified by using primers spanning sgRNA target sites, and cleaned by passing from PCR cleaning columns. Then, these samples from wild-type and mutants were mixed and denatured and re-annealed. Mismatches between wildtype and mutant PCR products are recognized by T7 endonuclease which generated double-strand breaks. Therefore mutations are presented by the fragmentation of PCR products compared to PCR products only from wildtype samples. (**b**) Full-length cDNAs from wildtype cell lines and knockout clones were amplified and cloned into an expression vector (pcDNA3.3xHA in frame) for sequencing and expression analysis. These vectors were transfected into HEK293T cells, and expression of wild-type and mutant *BMAL1* from our knockouts were analysed with anti-HA tag blotting. Full-length BMAL1 protein

from wildtype cells were shown by red arrows. T1 and T2 cDNAs expressed short proteins at very low level (**c**). Premature stop codons truncated proteins in the bHLH, and PAS domain was not expressed in the knockouts. Identification of CRISPR/Cas9 induced mutations in *BMAL1* gene by CRISP-ID (Detecting CRISPR mediated indels by Sanger sequencing) method (**d**). The alignment of the mutations in *BMAL1* knockouts. Green highlight: PAM Sequences, Yellow highlight: sgRNA target sequence, - premature stop codon caused by frameshift. MW: Molecular Weight in kDa; ** indicates the codon number where truncated proteins loose any significant homology with wildtype protein. MW calculation is based on the expressed proteins including frame shift caused peptides (maximum 10 a.a.) before the early stop codons.

Figure S2. Determination of time kinetic of c-PARP in MCF10A and MDA-MB-231 cells. (**a, b**) We searched the literature to find out suitable dose range of cisplatin and doxorubicin for the apoptotic responses of MCF10A and MDA-MB-231 cells to these genotoxic agents. We then analyzed the apoptotic response by quantitating the c-PARP signal over actin signal and by plotting this ratio over time. We selected the 16 hour-treatment for apoptosis assays on the fact that 16 hour-treatment showed a linear response. However, doses of drugs were further adjusted to get a linear response also with highly sensitized *BMAL1* knockout cells while also seeing a response with parental cells as shown in the main text figures. Numbers and marks on the left of each figure indicate the positions of the corresponding molecular size markers in kDa. Full-length blots are presented in Supplementary Figure S13.

Figure S3. Representative images of the second lines (L2s) of *BMAL1*-T1 and T2 knockouts showing the effect of *BMAL1* mutation on apoptosis induced by cisplatin and doxorubicin agents in *p53* WT cell line MCF10A. Due to space limitation, a representative image of apoptosis assay of only L1s (subclones of T1 and T2 sgRNA targeting) was shown in the main article (Fig. 2a and 2b), and the results of the other two lines (L2s) were shown here. *CRY*-DKO cell line was used as a control in addition to parental cell line to confirm that the amplification of apoptosis is not caused by procedures used to obtain knockouts. *CRY*-DKO mutation did not cause any effect on apoptosis following cisplatin or doxorubicin treatment. (**a, b**) Cells were treated with 0, 10, and 20 μmol/L cisplatin or 0, 0.5, and 1 μmol/L doxorubicin for 16 hours. Cell lysates were probed for cleaved PARP (c-PARP) by immunoblotting. Actin served as a loading control. The levels of c-

PARP from this image were included in the quantitation which was shown in the main text Fig. 2c. Numbers and marks on the left of each figure indicate the positions of the corresponding molecular size markers in kDa. Full-length blots are presented in Supplementary Figure S14.

Figure S4. Representative images of the second lines (L2s) of *BMAL1*-T1 and T2 knockouts showing the effect of *BMAL1* mutation on apoptosis induced by cisplatin and doxorubicin agents in *p53* mutant cell line MDA-MB-231. Due to space limitation, a representative image of apoptosis assay of only L1s (subclones of T1 and T2 sgRNA targeting) was shown in the main article (Fig. 3a and 3b), and the results of the other two lines (L2s) were shown here. A mock cell line of MDA-MB-231 was used a control in addition to parental cell line to confirm that the amplification of apoptosis is not caused by procedures used to obtain knockouts. (**a, b**) Cells were treated with 0, 20, and 40 μmol/L cisplatin or 0, 2.5, and 5 μmol/L doxorubicin for 16 hours. Cell lysates were probed for cleaved PARP (c-PARP) by immunoblotting. Actin served as a loading control. The levels of c-PARP from this image were included in the quantitation which was shown in the main text Fig. 3c. (**c**) *CRY*-DKO mutation, as expected, amplify cisplatin-induced apoptosis in this cell because of *p53* mutation in these cell lines as in agreement with our previous findings (See main text for the detailed explanation). (**d**) Therefore, *CRY*-DKO subclone of MDA-MB-231 cells displayed a higher level of c-PARP compared to parental cell lines. The knockout of *CRY*s were confirmed with western blotting. Numbers and marks on the left of each figure indicate the positions of the corresponding molecular size markers in kDa. KO indicates *BMAL1* knockouts generated by T1 or T2 sgRNA targeting, L indicates subclone for each sgRNA targeting. Full-length blots are presented in Supplementary Figure S15, which was divided into two parts as Supplementary Figure S15a and Supplementary Figure S15b.

Figure S5. The representative images of p53 immunoblotting of L2 subclones after treating cells with cisplatin or doxorubicin agents. Due to space limitation, representative images of p53 immunoblots of only L1 subclones were shown in the main article (Fig. 4a and 3b), and the results of the other two lines (L2s) were shown here. (**a, b**) MCF10A and MDA-MB-231 cells express wildtype and mutant p53 proteins, respectively. *BMAL1* mutation causes an amplification on apoptosis in both cell lines while wildtype p53 accumulates at similar levels in wildtype and *BMAL1* (as well as *CRY*) knockouts in MCF10A clones, which suggest that p53 does not explain

the amplification of apoptosis in *BMAL1* knockouts. Moreover, p53 is accumulated in MDA-MB-231 cells and its clones because of mutant *p53*. A mock (transduced with *BMAL1* targeting lentivirus but had a wildtype *BMAL1*) subclone of MDA-MB-231 was used as a control instead of *CRY*-DKO, because *CRY*-DKO in *p53* mutant background caused an amplification of cisplatin induced apoptosis in MDA-MB-231 cells in agreement with previous reports. Calnexin (CNX) served as a loading control. Numbers and marks on the left of each figure indicate the positions of the corresponding molecular size markers in kDa. KO indicates *BMAL1* knockouts generated by T1 or T2 sgRNA targeting, L indicates subclone for each sgRNA targeting. Full-length blots are presented in Supplementary Figure S16, which was divided into two parts as Supplementary Figure S16a and Supplementary Figure S16b.

Figure S6. Population Doubling (PD) Time of cell lines. PD times were calculated and compared to parental cell lines. PD times were not significantly altered by *BMAL1* or *CRY* mutations. KO indicates *BMAL1* knockouts generated by T1 or T2 sgRNA targeting, L indicates subclone for each sgRNA targeting.

Figure S7. Analysis of mycoplasma status of cell lines. No mycoplasma were contaminated in the cell lines used in this study. Full-length gels are presented in Supplementary Figure S17.

Figure S8-S11: Uncropped scans of blots, which were displayed in the main figures. Black dashed lines were added on raw images to indicate that two different membranes were imaged at the same time where it is not easy to notice.

Figure S12-S17: Uncropped scans of blots and gels, which were displayed in the Supplementary Figures S1-S5 and S7. Black dashed lines were added on raw images to indicate that two different membranes were imaged at the same time where it is not easy to notice.

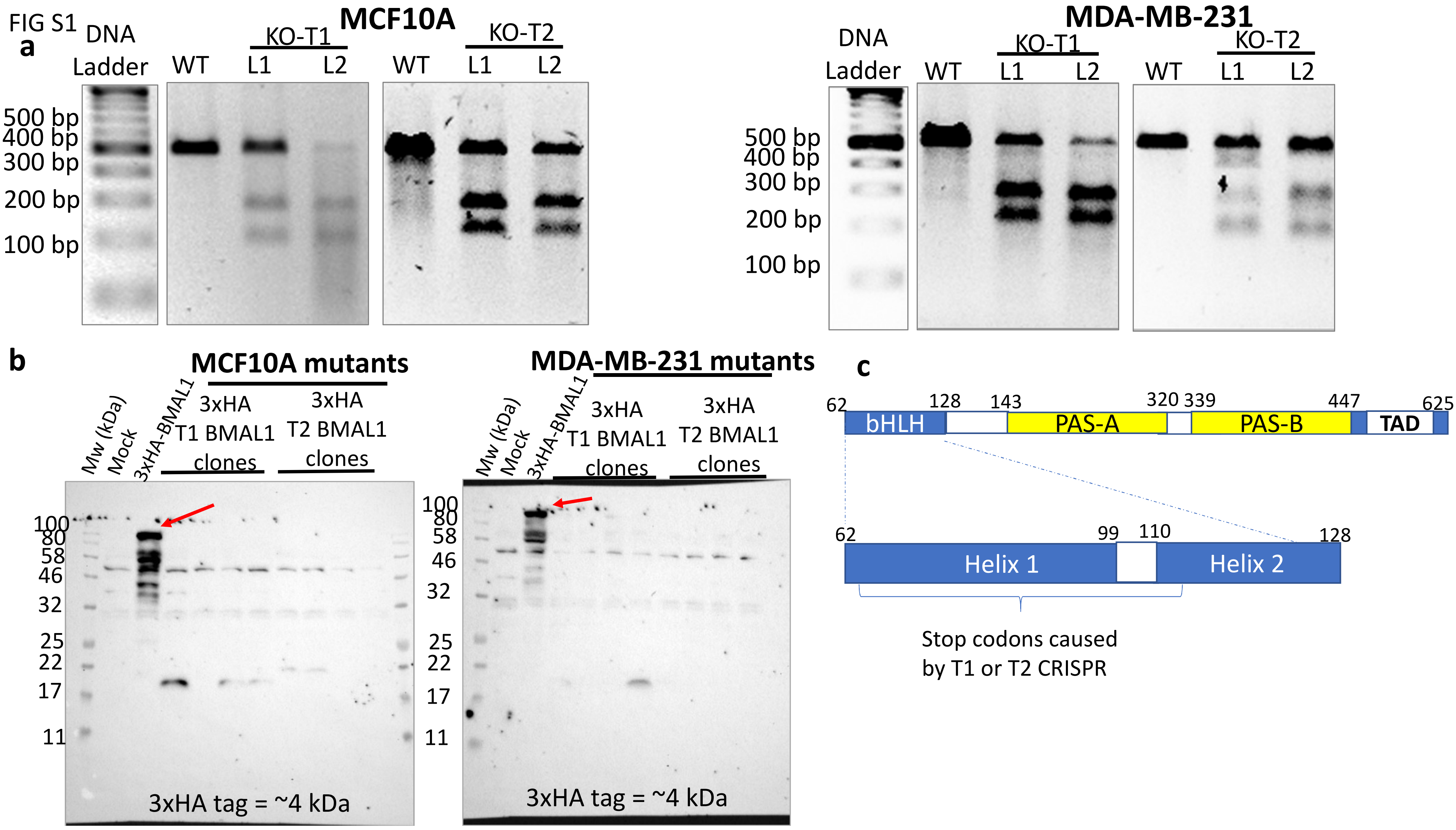
FIG S1
a
MCF10A
DNA Ladder
WT
KO-T1
L1
L2
WT
KO-T2
L1
L2
500 bp
400 bp
300 bp
200 bp
100 bp
MDA-MB-231
DNA Ladder
WT
KO-T1
L1
L2
WT
KO-T2
L1
L2
500 bp
400 bp
300 bp
200 bp
100 bp
b
MCF10A mutants
Mw (kDa)
Mock
3xHA-BMAL1
3xHA T1 BMAL1 clones
3xHA T2 BMAL1 clones
100
80
58
46
32
25
22
17
11
3xHA tag = ~4 kDa
MDA-MB-231 mutants
Mw (kDa)
Mock
3xHA-BMAL1
3xHA T1 BMAL1 clones
3xHA T2 BMAL1 clones
100
80
58
46
32
25
22
17
11
3xHA tag = ~4 kDa
c
62
128
143
320
339
447
625
bHLH
PAS-A
PAS-B
TAD
62
99
110
128
Helix 1
Helix 2
Stop codons caused by T1 or T2 CRISPR

Figure S1d

T1 sgRNA-induced knockout sequencing

MCF10A T1 (L2 line)

CRISP-ID alignment from amplified cDNA sanger sequencing

```
    1 CCGGGCCCCACCGACCTGCTTTCCAGCTCTCTTGGTACCAGTGGTGTGGATTGCAACCGCAAACGGAAAGGCAGCTCCACTGACTACCAAGAAAGCATGG  100
    1 --GGGCCCCACCGACCTGCTTTCCAGCTCTCTTGGTACCAGTGGTGTGGATTGCAACCGCAAACGGAAAGGCAGCTCCACTGACTACCAAGAAAGCATGG   98
    1 --GGGCCCCACCGACCTGCTTTCCAGCTCTCTTGGTACCAGTGGTGTGGATTGCAACCGCAAACGGAAAGGCAGCTCCACTGACTACCAAGAAAGCATGG   98

      101                                                                                              200
  101 ACACAGACAAAGATGACCCTCATGGAAGGTTAGAATATACAGAACACCAAGGAAGGATAAAAAATGCAAGGGAAGCTCACAGTCAGATTGAAAAGCGGCG  200
   99 ACACAGACAAAGATGACCCTCAGGGAAGG----AATATACAGAACACCAAGGAAGGATAAAAAATGCAAGGGAAGCTCACAGTCAGATTGAAAAGCGGCG  193
   99 ACACAGACAAAGATGACCCTCATGGAAGG-------ATACACAACACCCCGGAGGGAGAAAAAATGCGCGGGAAACTCACAGTCACATTGAAAAGCGGCG  191

      201                                                                                              300
  201 TCGGGATAAAATGAACAGTTTTATAGATGAATTGGCTTCTTTGGTACCAACATGCAACGCAATGTCCAGGAAATTAGATAAACTTACTGTGCTAAGGATG  300
  194 TCGGGATAAAATGAACAGTTTTATAGATGAATTGGCTTCTTTGGTACCAACATGCAACGCAATGTCCAGGAAATTAGATAAACTTACTGTGCTAAGGATG  293
  192 TCGGGATAAAATGAACAGTTTTATAGATGAATTGTCTTCTTTGGGACCAACATGCGACGCACTGTCCAGGAAATTATATAAACTTACTGTGCTAAGGAGG  291
```

Corresponding Protein Sequences and calculated MWs:

Wildtype Protein
..GPTDLLSSSLGTSGVDCNRKRKGSSTDYQESMDTDKDDPHGRLEYTEHQGRIKNAREAHSQIEKRRRDKMNSFIDELASLV….
MW: 68.69 kDa

Protein 1
…GPTDLLSSSLGTSGVDCNRKRKGSSTDYQESMDTDKDDPQGRNIQNTKEG-
MW: 8.26 kDa (Codon 60**)

Protein 2
…GPTDLLSSSLGTSGVDCNRKRKGSSTDYQESMEQTKMTLMEGYTTPRREKKCAGNSQSH-
MW: 9.34 (Codon 58**)

MDA-MB-231 T1 (L2 line)

CRISP-ID Alignment from cDNA sequence

```
   1 --GTGGATTGCAACCGCAAACGGAAAGGCAGCTCCACTGACTACCAAGAAAGCATGGACACAGACAAAGATGACCCTCATGGAAGGTTAGAATATACAGA   98
   1 GTGTGGATTGCAACCGCAAACGGAAAGGCAGCTCCACTGACTACCAAGAAAGCATGGACACAGACAAAGATGACCCTCATGGAAG-----AATATACCGA   95
   1 GTGTGGATTGCAACCGCAAACGGAAAGGCACCTCCCCTGACTACCAAAAAAGCGGGGACCCAAACAAAGATGACCCTCAGGGAAA-----AATATACAGA   95

     101                                                                                185
  99 ACACCAAGGAAGGATAAAAAATGCAAGGGAAGCTCACAGTCAGATTGAAAAGCGGCGTCGGGATAAAATGAACAGTTTTATAGAT 183
  96 ACACCAGGGAAGGATAAAAAATGCAAGGGAAGCTCACAGTCAAATTGAAAAGCGGCGTCGGGATAAAATGAACAGTTTTATA--A 178
  96 ACACCAAGGAAGGATAAAAAATGCAAGGGAAGCTCACAGTCAGATTGAAAAGCGGCGTCAGGATAAAATGAACAGTTTTATAG—178
```

Corresponding Protein Sequences and calculated MWs:

Wildtype Protein
…VDCNRKRKGSSTDYQESMDTDKDDPHGRLEYTEHQGRIKNAREAHSQIEKRRRDKMNS…
MW: 68.69 kDa

Protein 1
…VDCNRKRKGSSTDYQESMDTDKDDPHGRIYRTPGKDKKCKGSSQSN-
MW: 8.5 kDa (Codon 64**)

Protein 2
…VDCNRKRKGTSPDYQKSGDPNKDDPQGKIYRTPRKDKKCKGSSQSD-
MW: 8.5 kDa (Codon 64**)

T2 sgRNA-induced knockout sequencing

MCF10A T2 (L2 line)

CRISP-ID alignment from amplified gDNA sanger sequencing (Reverse reading from gDNA)

```
   1 GCCTTAGGGATAGGAACATTCTAGGCAAGGGTGGTAACATTTGTGGGCAAGGACATAAAGGACAATAGAGCCCAGGGTCTCACCTCTTAATGTTTTCATG 100
   1 GCCTTAGGGATAGGAACATTCTAGGCAAGGGTGGTAACATTTGTGGGCAAGGACATAAAGGACAATAGAGCCCAGGGTCTCACCTCTTAATGTTTTCATG 100
   1 GCCTTAGGGATAGGAACATTCTAGGCAAGGGTGGTAACATTTGTGGGCAAGGACATAAAGGACAATAGAGCCCAGGGTCTCACCTCTTAATGTTTTCATG 100
```

Figure S1d continued

```
 101 TGCTGAACAGCCATCCTTAGCACAGTAAGTTTATCTAATTTCCTGGACATTGCGTTGCATGTTGGTACCAAAGAAGCCAATTCATCTATAAAACTGTTCA 200
 101 TG-------------------------------------------GACATTGCGTTGCATGTTGGTACCAAAGAAGCCAATTCATCTATAAAACTGTTCA 157
 101 TGCTGAACAGCCATCCTT---------------------------------------------------------------------------------- 118

 201 TTTTATCCCGACGCCGCTTTTCAATCTGACTGTGAGCTTCCCTGAACAACAATATGAAAAACGATTTCTTATAAAATGTAACCAAGCATCAAGAAAAGAT 300
 158 TTTTATCCCGACGCCGCTTTTCAATCTGACTGTGAGCTTCCCTGAACAACAATATGAAAAACGATTTCTTATAAAATGTAACCAAGCATCAAGAAAAGAT 257
 118 -------------------TTCATTCTGACTGTGAGCTTCCCTGAACAACAATATGAAAAACGATTTCTTATAAAATGTAACCAAGCATCAAAAATAGAC 199

 301 GACAAAAATAAATGAGTTGT
 258 GACAAAAATAAATGAGTTGT
 200 GGAACAAATATATGAGTTGT
```

Corresponding Protein Sequences and calculated MWs:

Wildtype Protein

…HSQIEKRRRDKMNSFIDELASLVPTCNAMSRKLDKLTVLRMAVQHMKTL…

MW: 68.69 kDa

Protein 1

...HSQIEKRRRDKMNSFIDELASLVPTCNAMST-

MW: 11.8 kDa (Codon 106**)

Protein 2

…HSQNEKDGCSAHENIKR-

MW: 10.361 kDa (Codon 82**)

MDA-MB-231 T2 (L2 line)

CRISP-ID alignment from amplified gDNA sanger sequencing (Reverse reading from gDNA)

```
   1 GCCTTAGGGATAGGAACATTCTAGGCAAGGGTGGTAACATTTGTGGGCAAGGACATAAAGGACAATAGAGCCCAGGGTCTCACCTCTTAATGTTTTCATG 100
   1 --CTTAGGGATAGGAACATTCTAGGCAAGGGTGGTAACATTTGTGGGCAAGGACATAAAGGACAATAGAGCCCAGGGTCTCACCTCTTAATGTTTTCATG  98
   1 --CTTAGGGATAGGAACATTCTAGGCAAGGGTGGTAACATTTGTGGGCAAGGACATAAAGGACAATAGAGCCCAGGGTCTCACCTCTTAATGTTTTCATG  98

 101 TGCTGAACAGCCATCCTTAGCACAGTAAGTTTATCTAATTTCCTGGACATTGCGTTGCATGTTGGTACCAAAGAAGCCAATTCATCTATAAAACTGTTCA 200
  99 TG-------------------------------------------GACATTGCGTTGCATGTTGGTACCAAAGAAGCCAATTCATCTATAAAACTGTTCA 155
  99 TGCAGA-------------------------------------------TTGCCCTGCCTGTTCGTACCGACTGTGACCATTCCTCTATAACACTGTGAA 155

 201 T-TTTATCCCGACGCCGCTTTTCAATCTGACTGTGAGCTTCCCTGAACAACAATATGAAAAACGATTTCTTATAAAATGTAACCAAGCATCAAGAAAAGA 299
 156 T-TTTATCCCGACGCCGCTTTTCAATCTGACTGTGAGCTTCCCTGAACAACAATATGAAAAACGATTTCTTATAAAATGTAACCAAGCATCAAGAAAAGA 254
 156 TAACAATCTC-ACACCACATGTCACCCAGAATGTGAACTTCCCTGAACAACAATATGAAAAACGATTTCTTATAAAATGTAACCGACCATCAAGAAAAGA 254

 300 TGACAAAAATAAATGAGTTGTAACTGTTTTCAATGTTCATCTGACAAGGCAAATTTAGAATTTGCAGAATGTTTAAAATTA 380
 255 TGACAAAAATAAATGAGTTGTAACTGTTTTCAATGTTCATCTGACAAGGCAAATTTAGAATTTGCAGAATGTTTAAAATTA 335
 255 AGACTGCAAAAAGTGAATTGTAACAGTTTTCAATGTTCATCTGACAAAGCAAATTTACAATTTGCAGAATGTTTAAAATTA 335
```

Corresponding Protein Sequences and calculated MWs:

Wildtype Protein

….HSQIEKRRRDKMNSFIDELASLVPTCNAMSRKLDKLTVLRMAVQHMKTL…

MW: 68.69 kDa

Protein 1

….HSQIEKRRRDKMNSFIDELASLVPTCNAMST-

MW: 11.8 kDa (Codon 106**)

Protein 2

….HSVIEEWSQSVRTGRAICT-

MW: 10.56 kDa (Codong 81**)

FIG S2

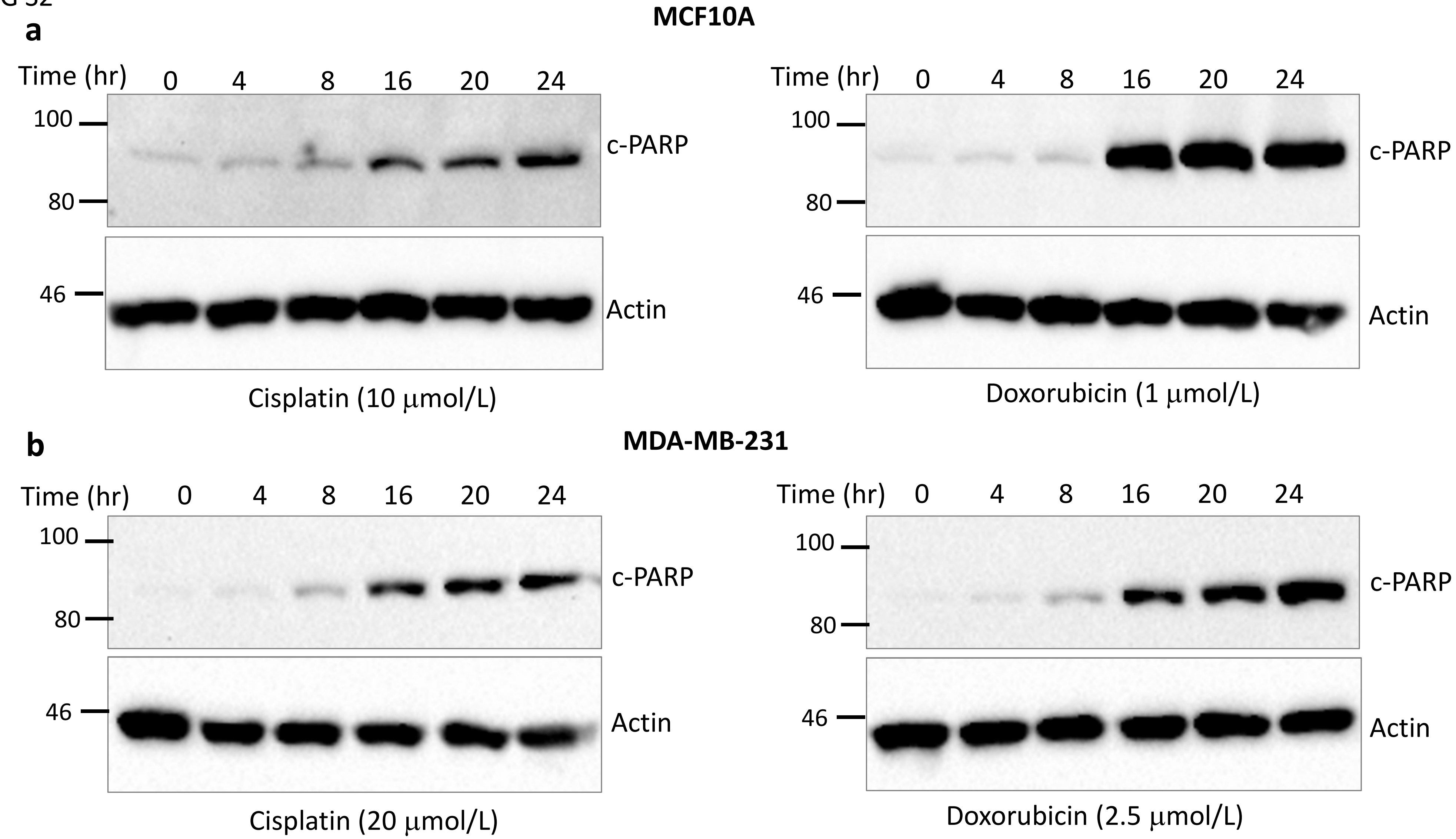

a
MCF10A
Time (hr) 0 4 8 16 20 24
100
80
46
c-PARP
Actin
Cisplatin (10 μmol/L)
Time (hr) 0 4 8 16 20 24
100
80
46
c-PARP
Actin
Doxorubicin (1 μmol/L)
b
MDA-MB-231
Time (hr) 0 4 8 16 20 24
100
80
46
c-PARP
Actin
Cisplatin (20 μmol/L)
Time (hr) 0 4 8 16 20 24
100
80
46
c-PARP
Actin
Doxorubicin (2.5 μmol/L)

FIG S3

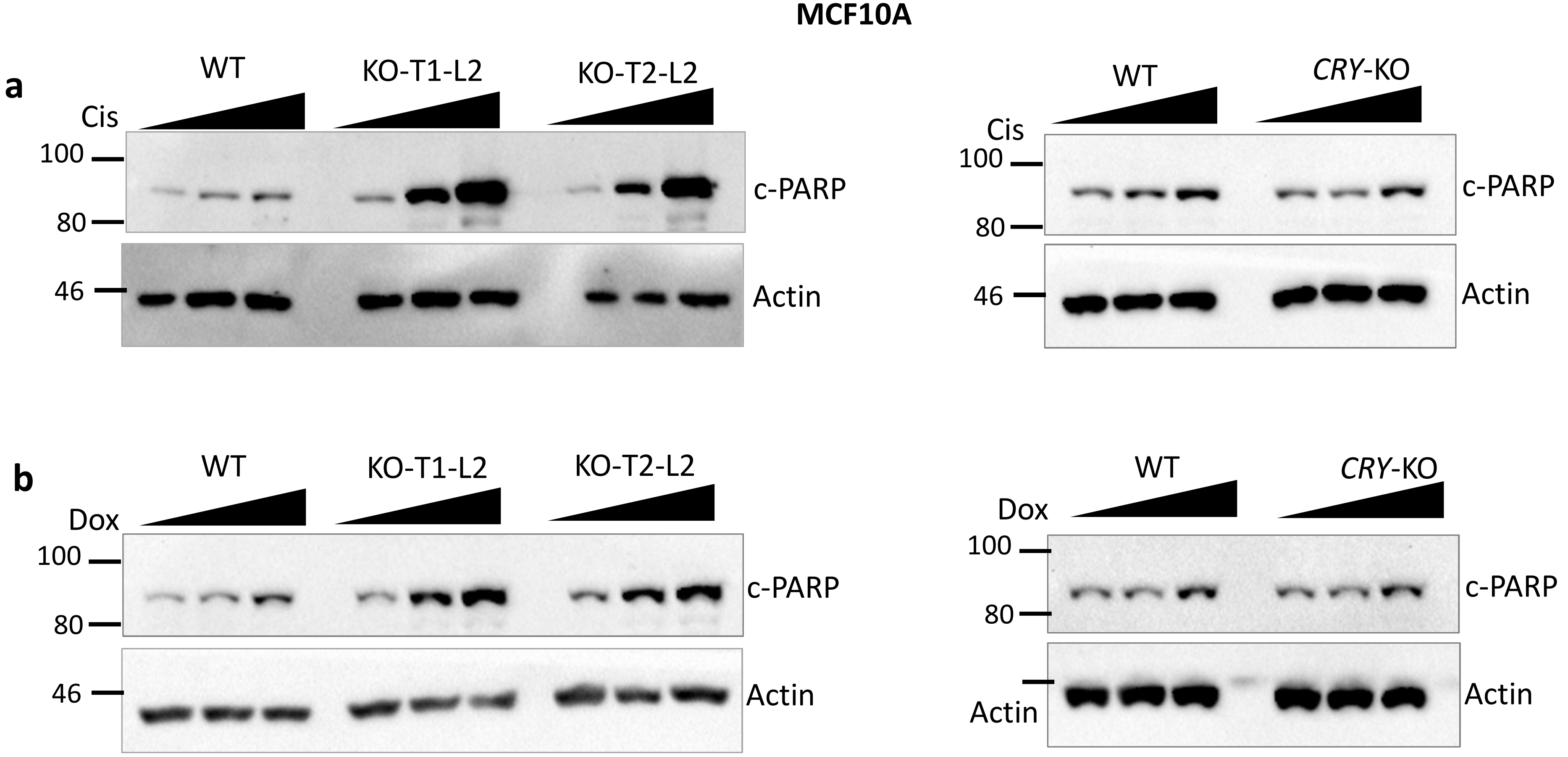
MCF10A
a
WT
KO-T1-L2
KO-T2-L2
Cis
100
80
c-PARP
46
Actin
WT
CRY-KO
Cis
100
80
c-PARP
46
Actin
b
WT
KO-T1-L2
KO-T2-L2
Dox
100
80
c-PARP
46
Actin
WT
CRY-KO
Dox
100
80
c-PARP
Actin
Actin

FIG S4

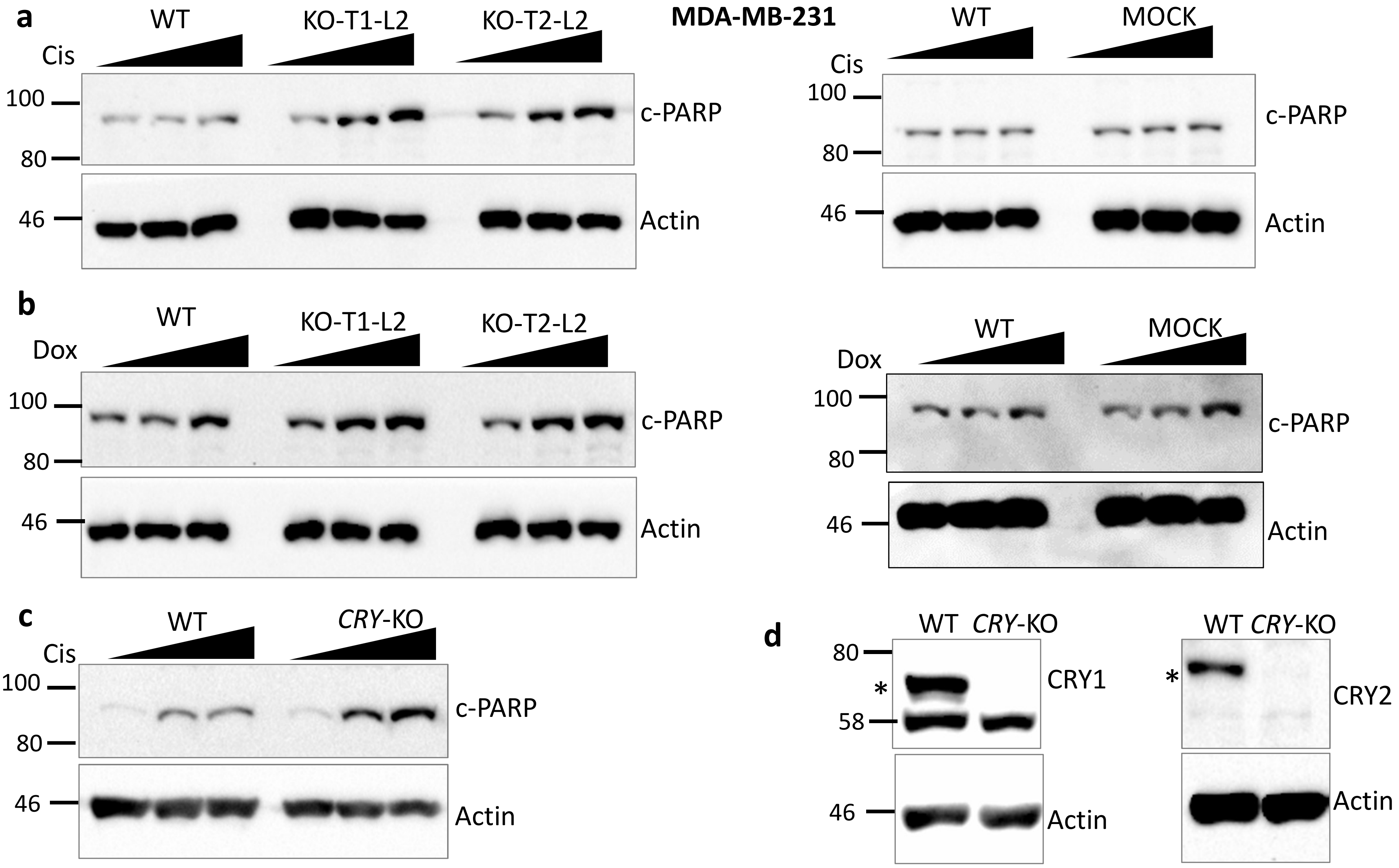
a
WT
KO-T1-L2
KO-T2-L2
MDA-MB-231
WT
MOCK
Cis
100
80
c-PARP
46
Actin
b
Dox
c
CRY-KO
d
58
CRY1
CRY2
*

FIG S5

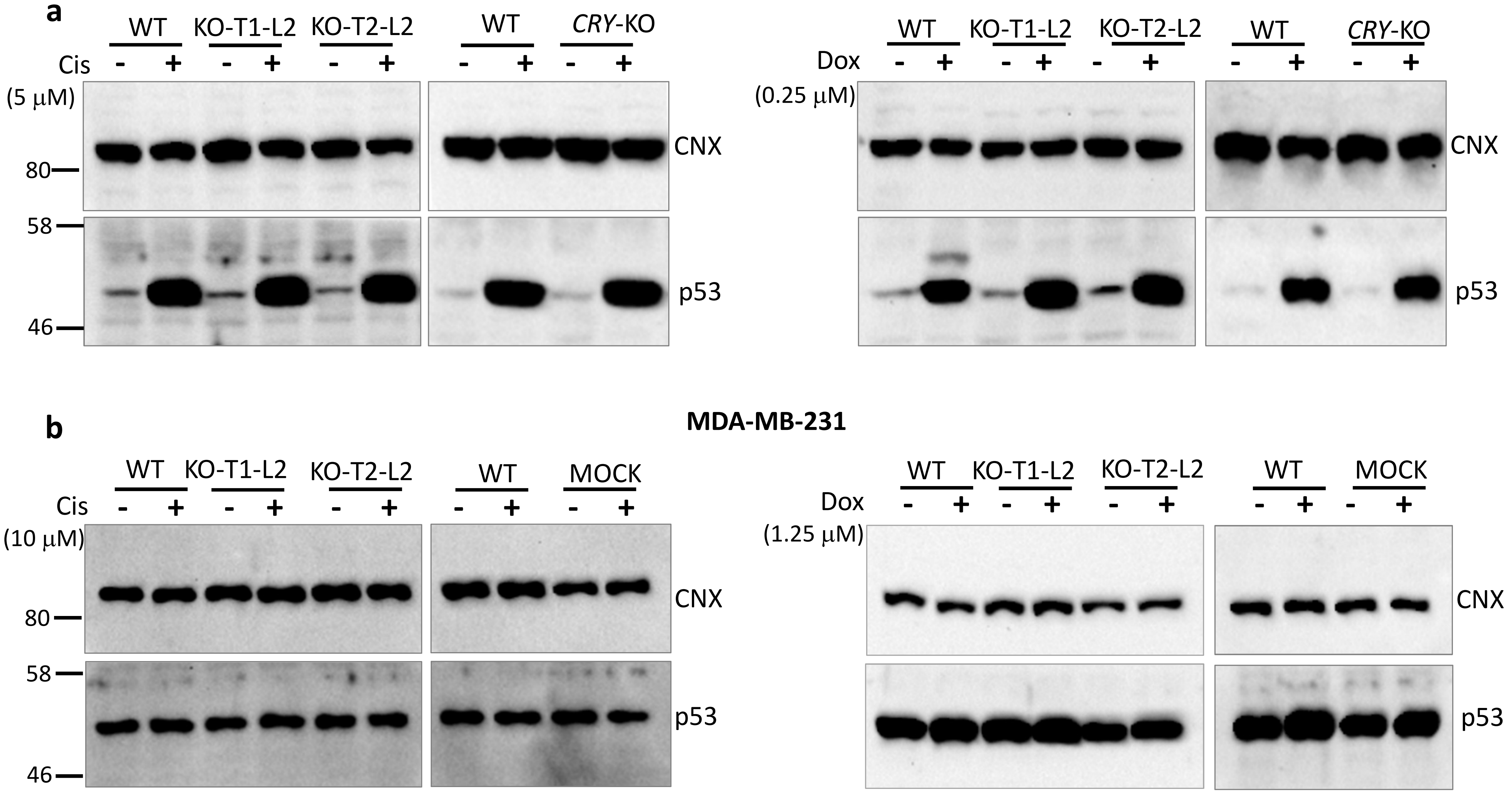
MCF10A
a
WT
KO-T1-L2
KO-T2-L2
WT
CRY-KO
Cis
(5 μM)
Dox
(0.25 μM)
CNX
p53
80
58
46
b
MDA-MB-231
MOCK
(10 μM)
(1.25 μM)

FIG S6

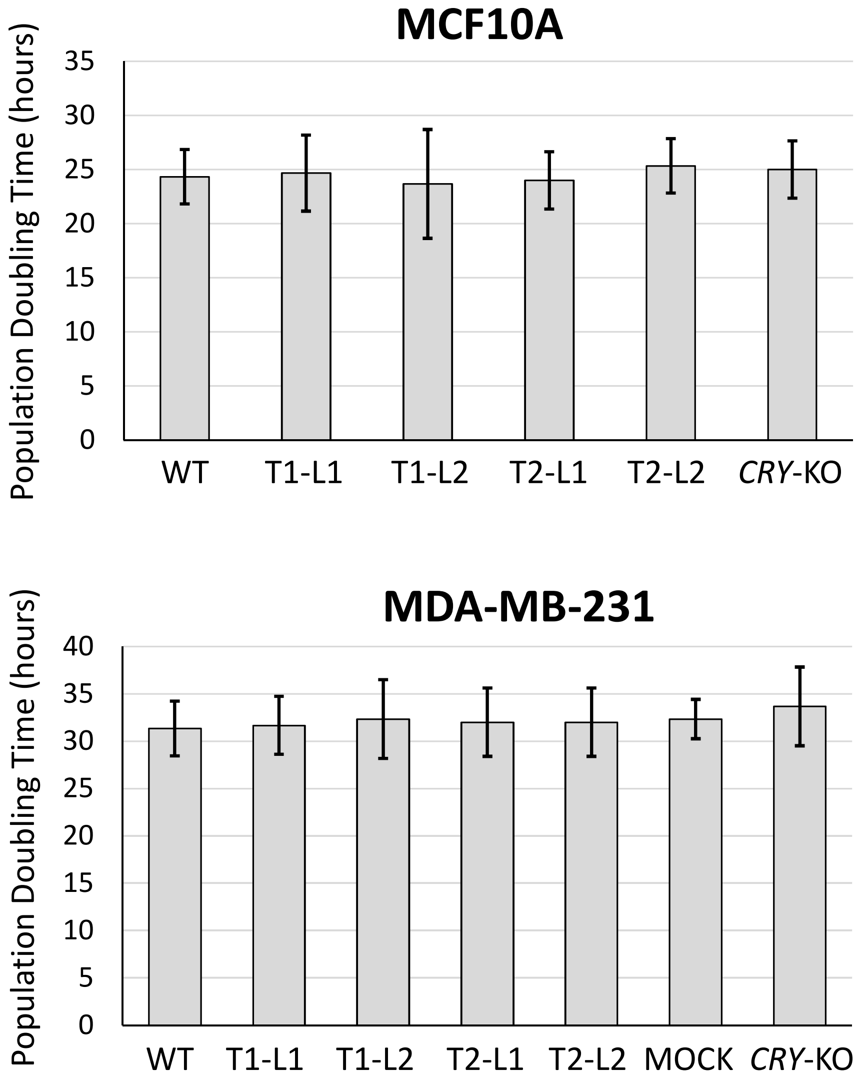
MCF10A
Population Doubling Time (hours)
35
30
25
20
15
10
5
0
WT
T1-L1
T1-L2
T2-L1
T2-L2
CRY-KO
MDA-MB-231
Population Doubling Time (hours)
40
35
30
25
20
15
10
5
0
WT
T1-L1
T1-L2
T2-L1
T2-L2
MOCK
CRY-KO

FIG S7

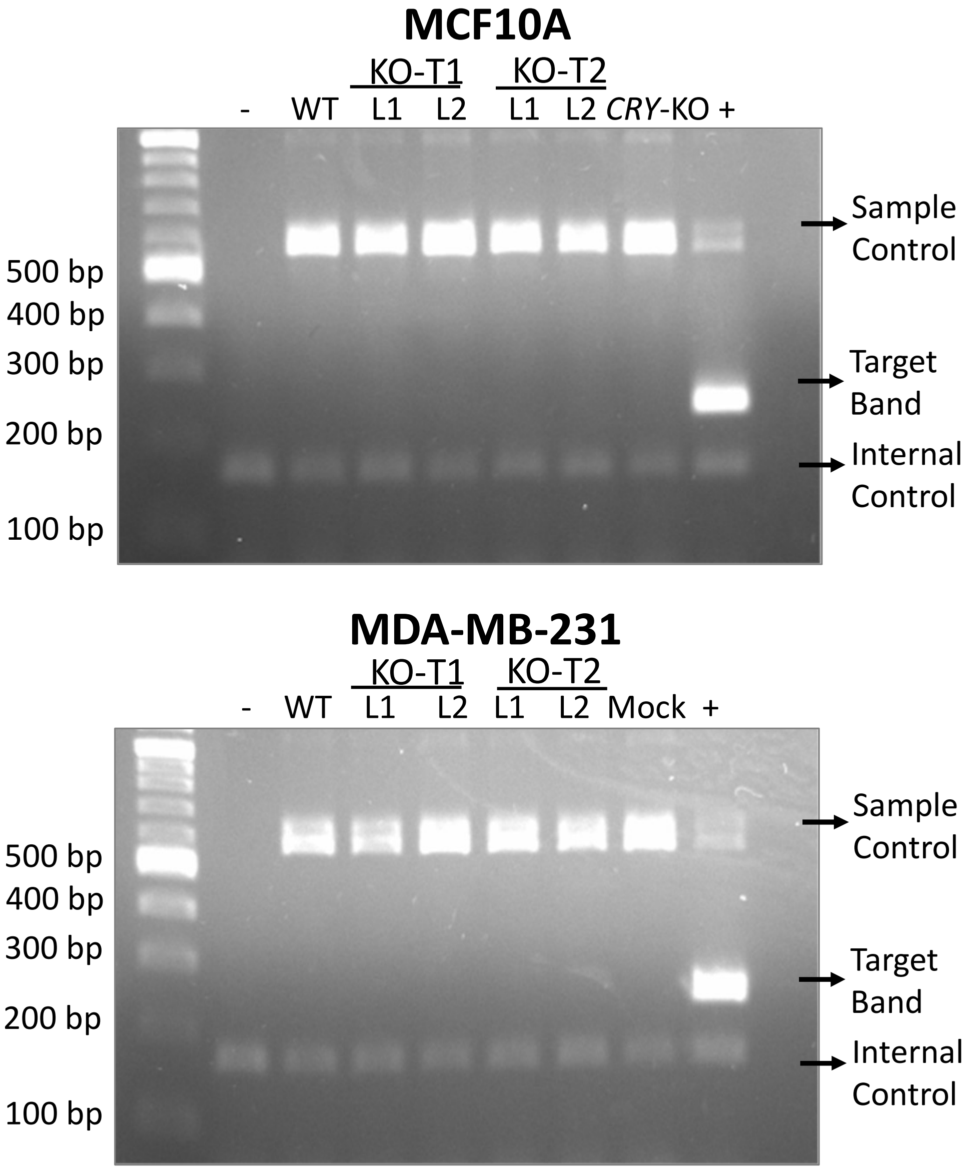

MCF10A
KO-T1
KO-T2
- WT L1 L2 L1 L2 CRY-KO +
500 bp
400 bp
300 bp
200 bp
100 bp
Sample Control
Target Band
Internal Control
MDA-MB-231
KO-T1
KO-T2
- WT L1 L2 L1 L2 Mock +
500 bp
400 bp
300 bp
200 bp
100 bp
Sample Control
Target Band
Internal Control

FIG S8

Fig 1a.

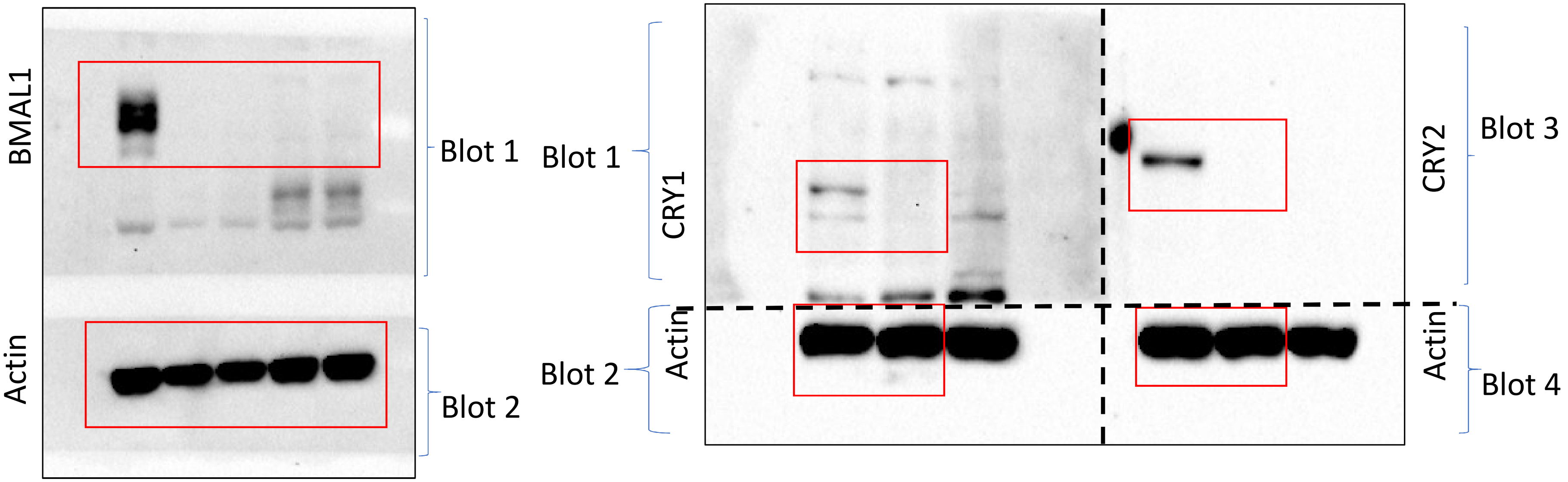

Fig 1b.

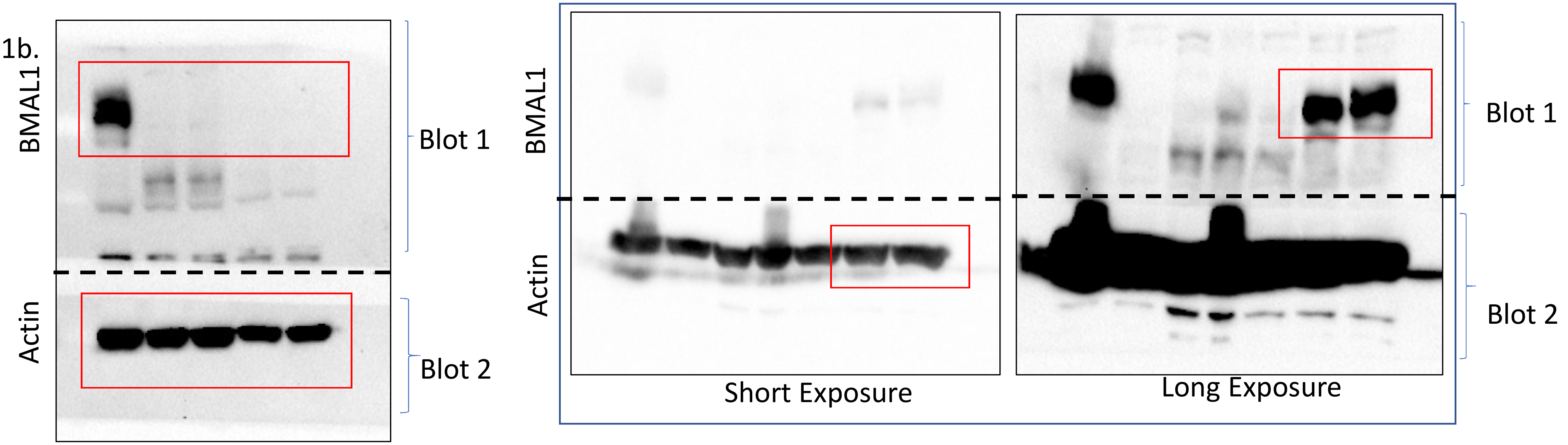

FIG S9

Fig 2a.

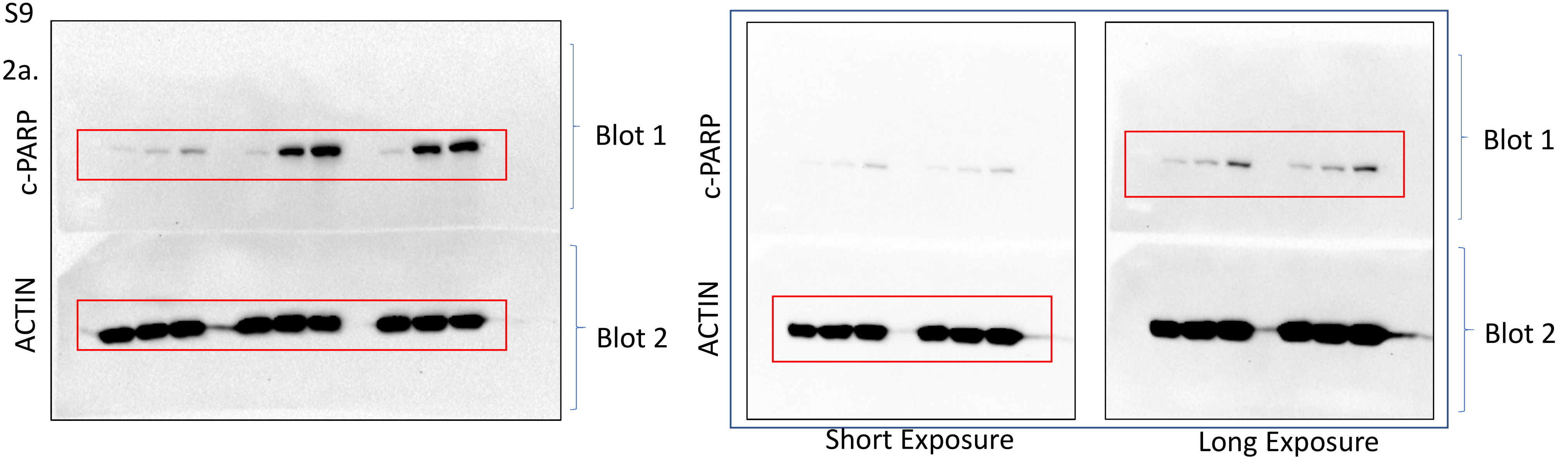

Fig 2b.

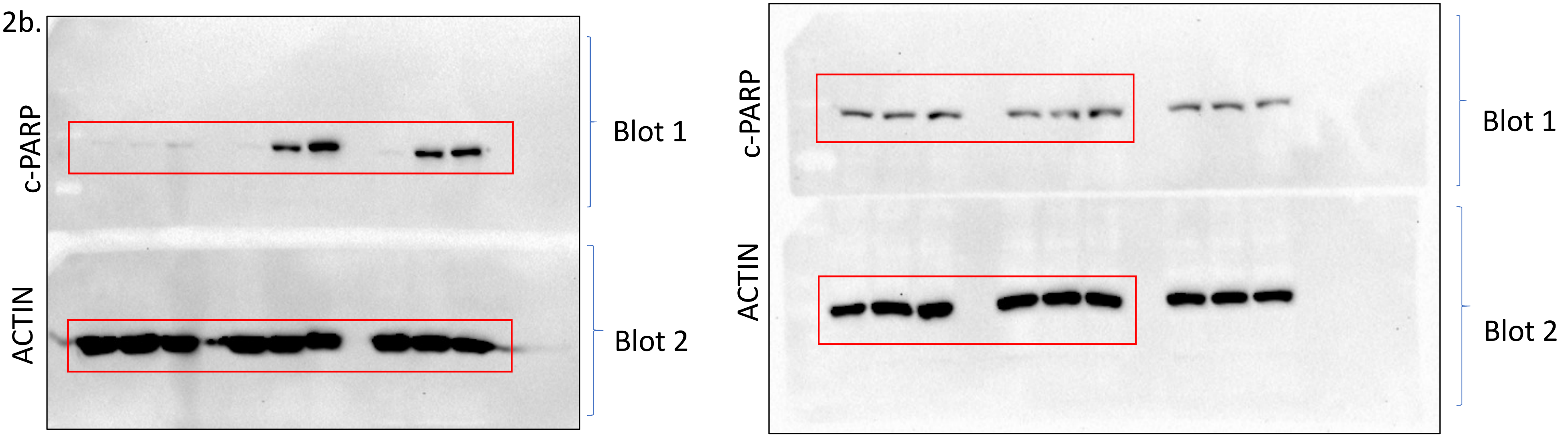

FIG S10

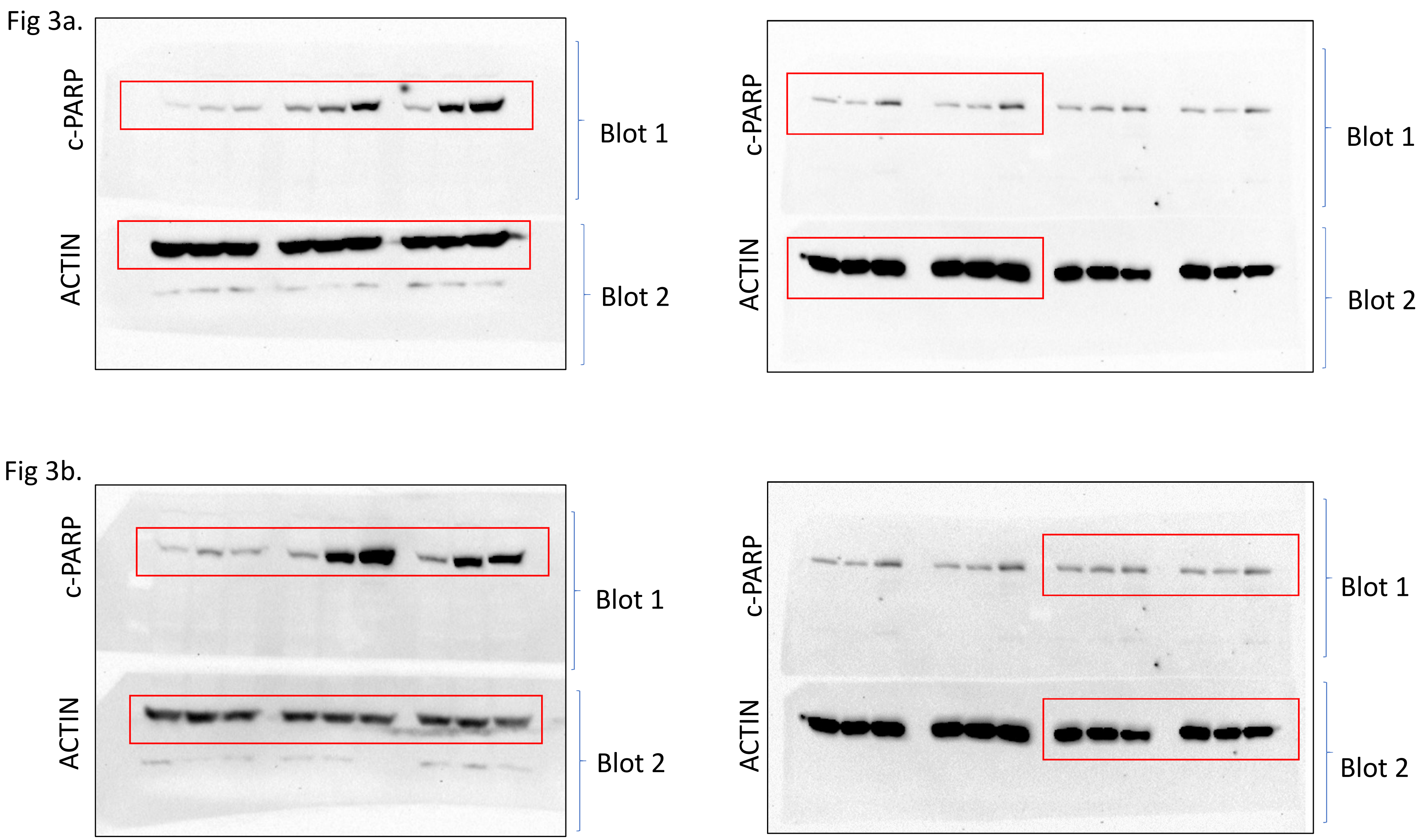
Fig 3a.
c-PARP
ACTIN
Blot 1
Blot 2
c-PARP
ACTIN
Blot 1
Blot 2
Fig 3b.
c-PARP
ACTIN
Blot 1
Blot 2
c-PARP
ACTIN
Blot 1
Blot 2

FIG S11

Fig 4a.

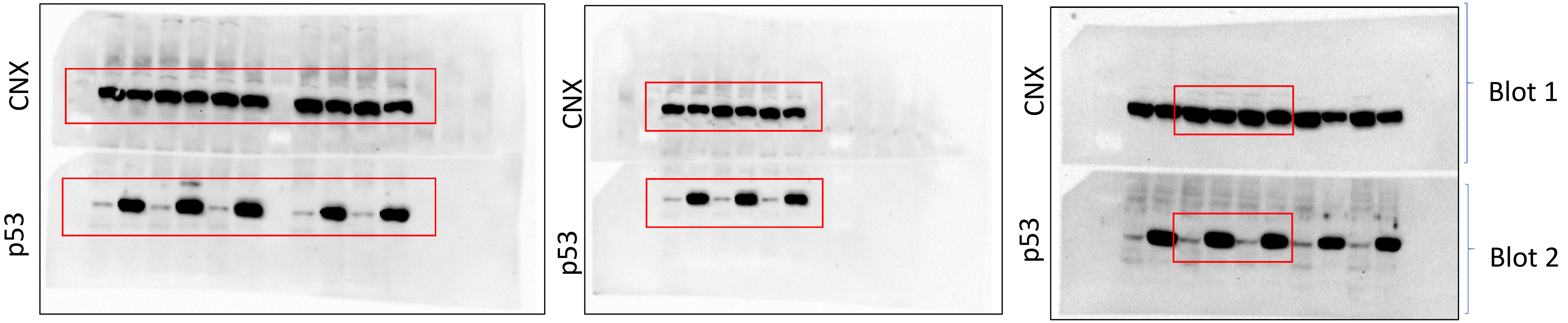

Fig 4b.

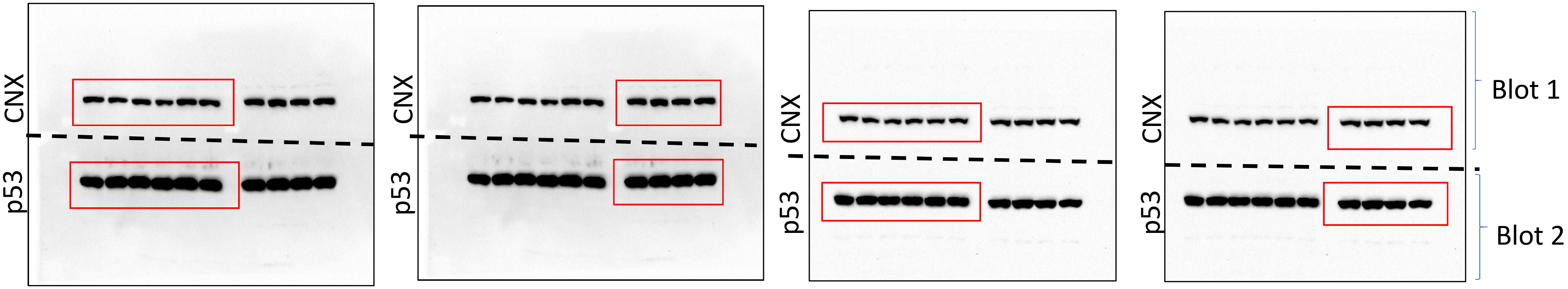

FIG S12a

MCF10A

Fig S1a.

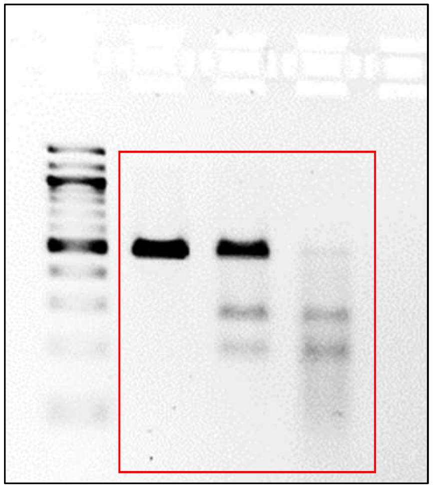

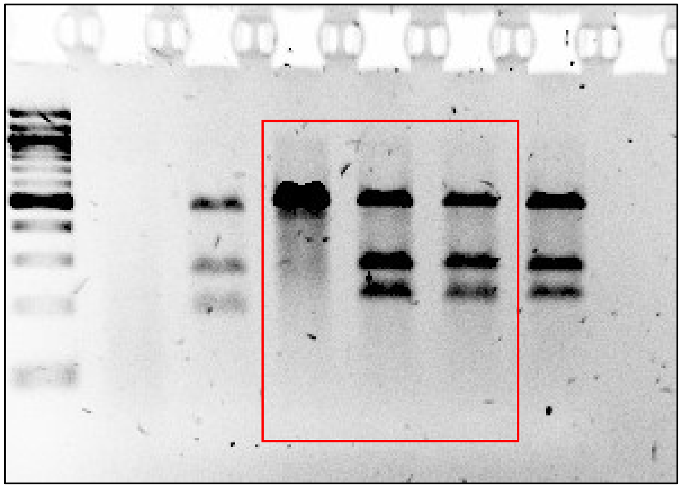

MDA-MB-231

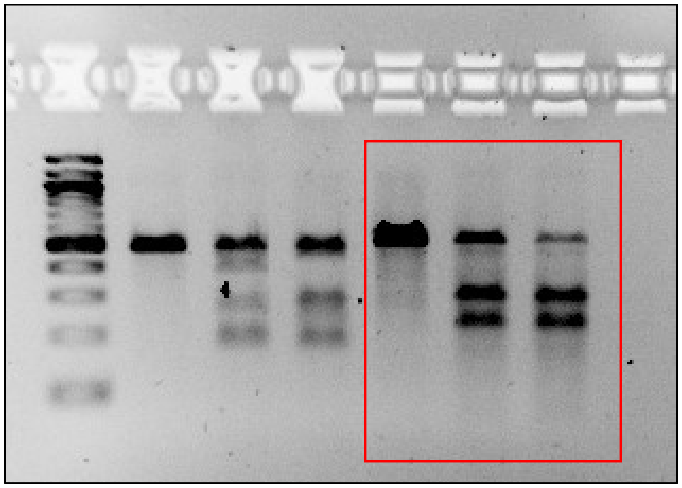

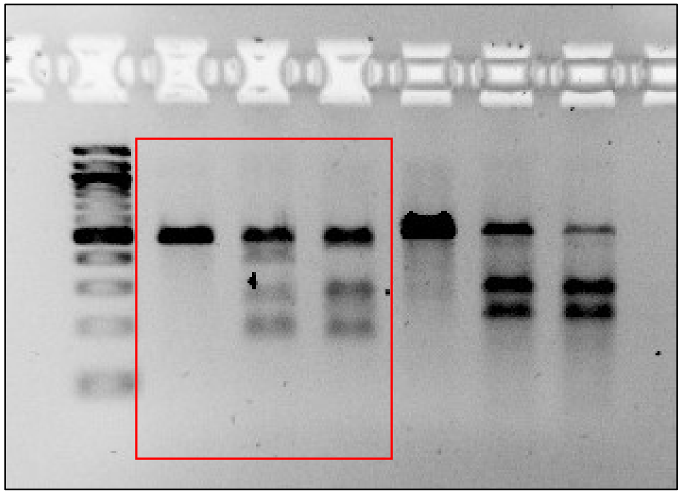

FIG S12b

FIG S1b

MCF10A mutants

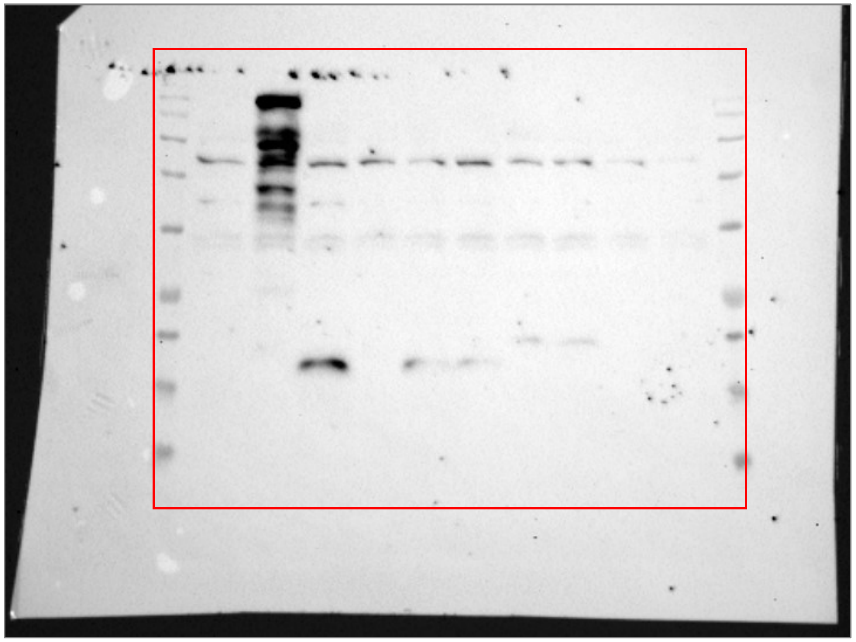

MDA-MB-231 mutants

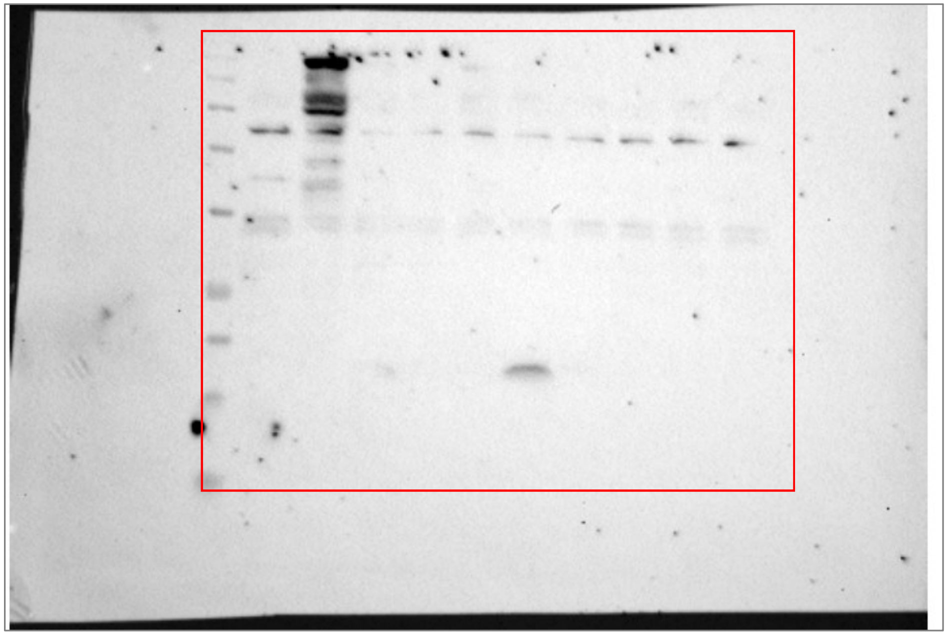

FIG S13

Fig S2a.

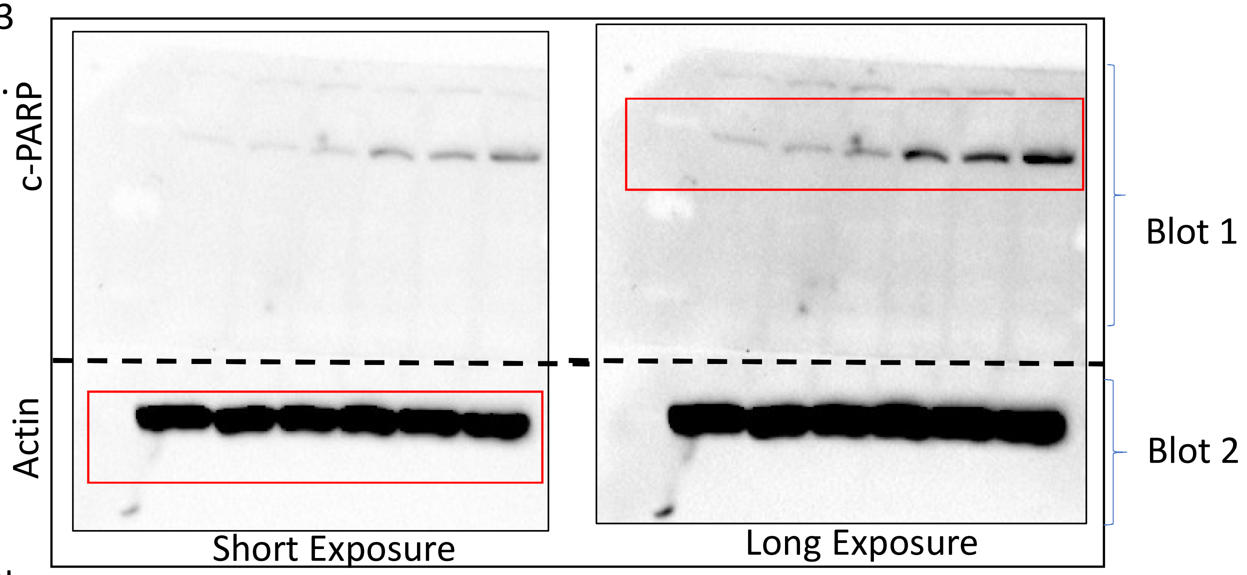

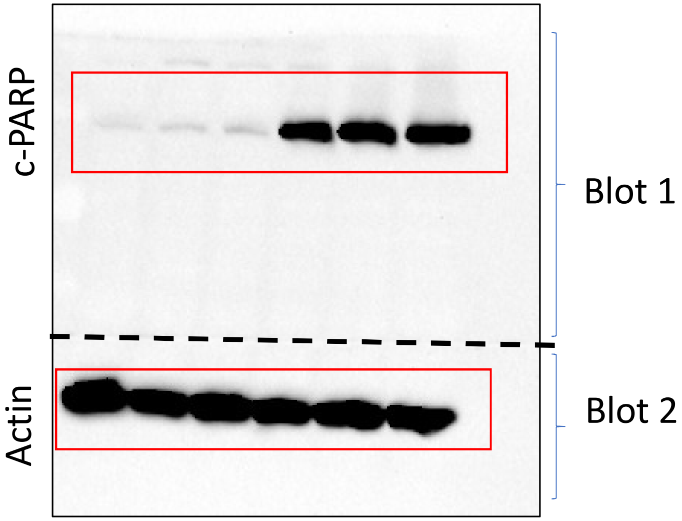

Fig S2b.

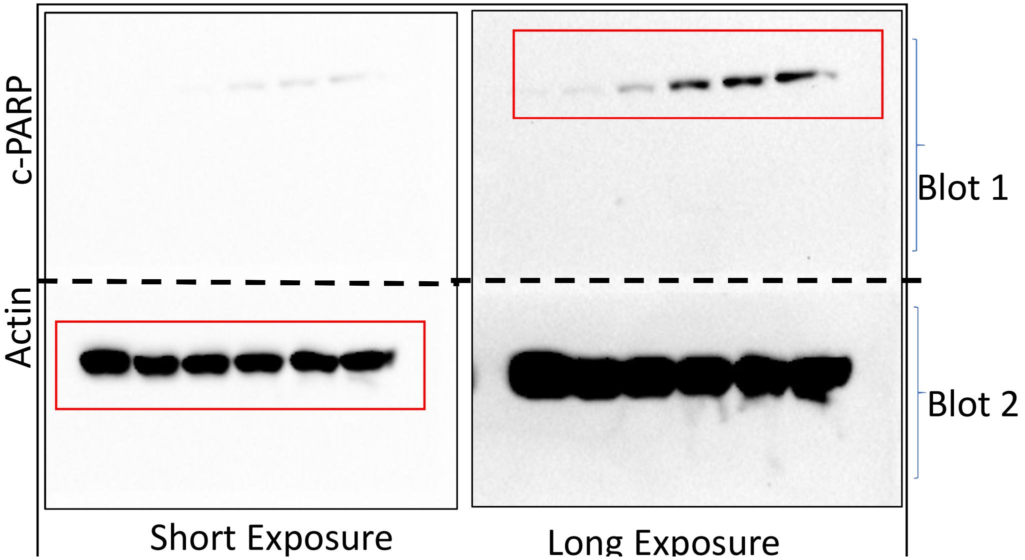

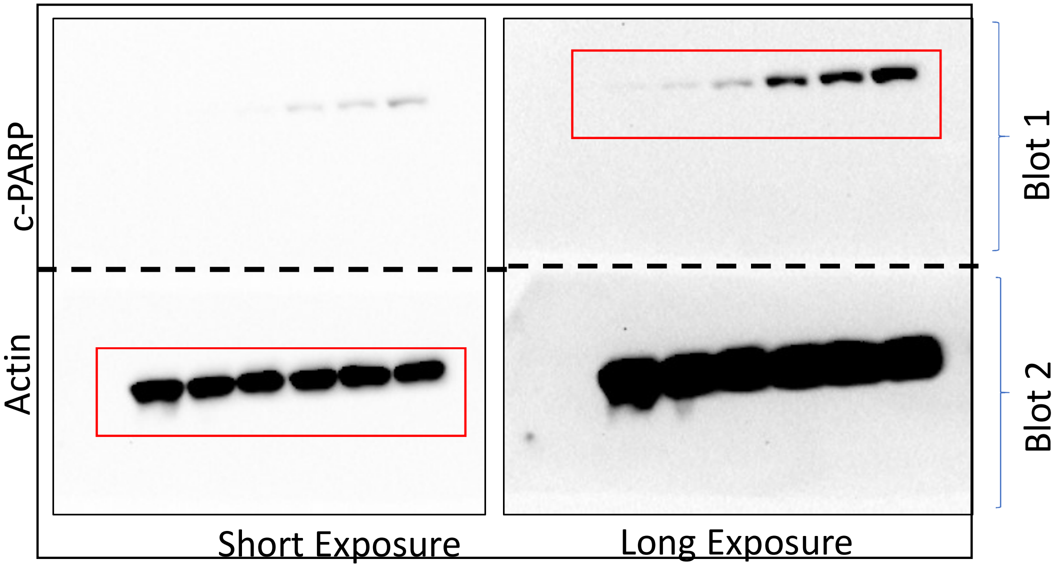

FIG S14

Fig S3a.

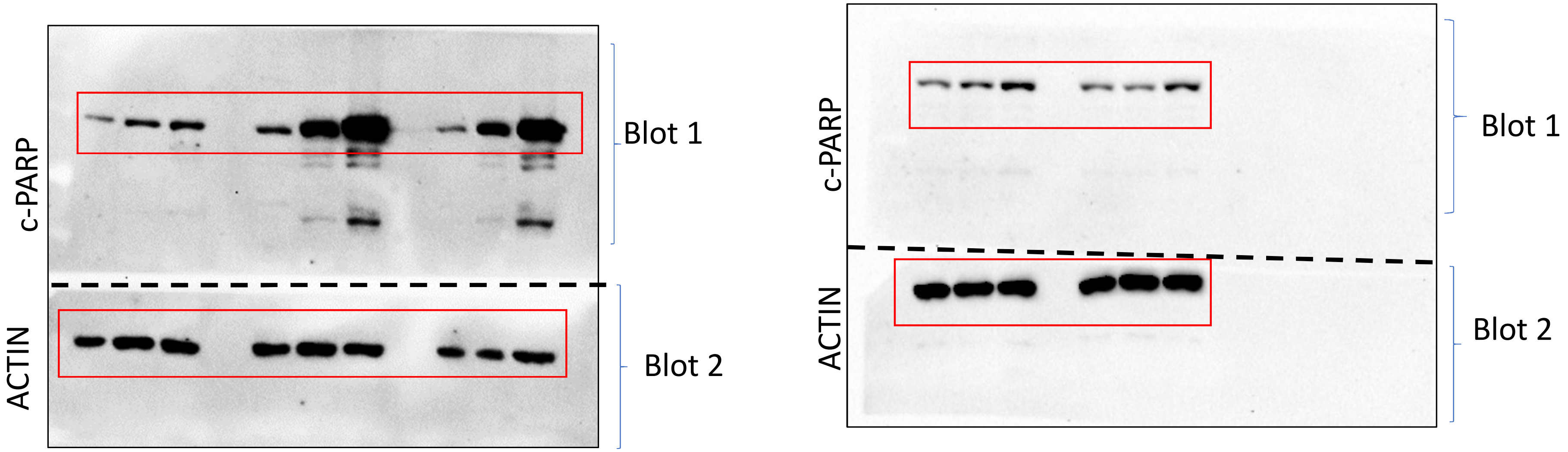

Fig S3b.

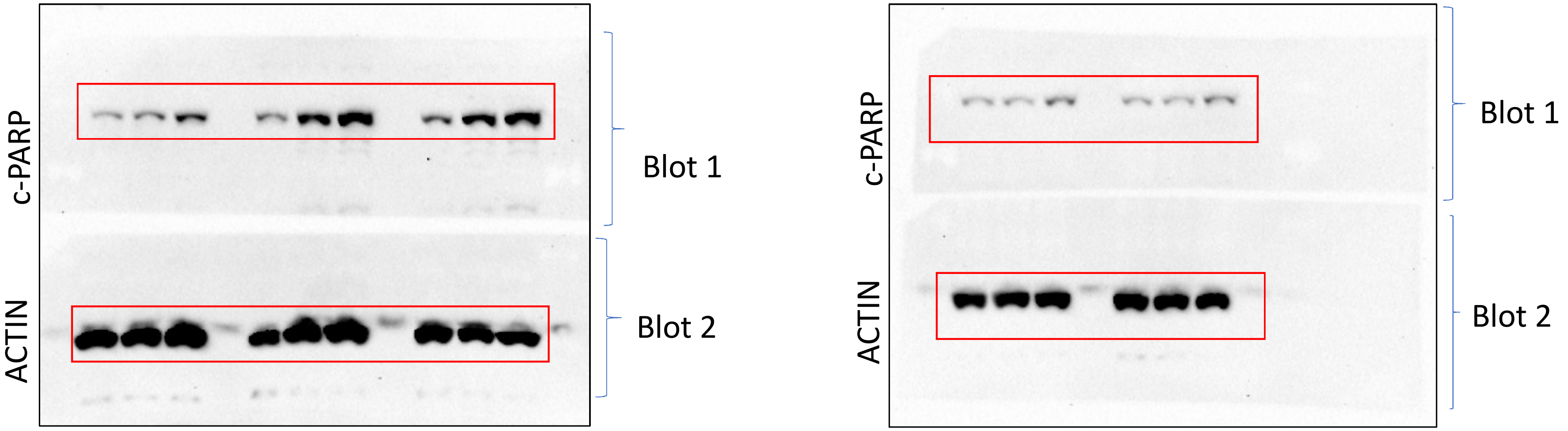

FIG S15a
Fig S4a.

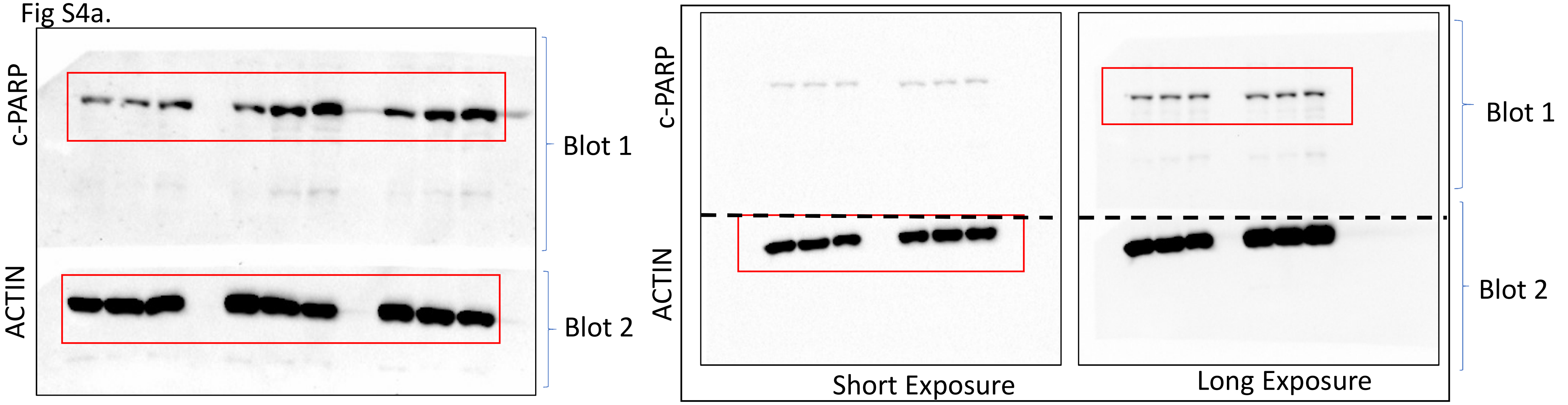

Fig S4b

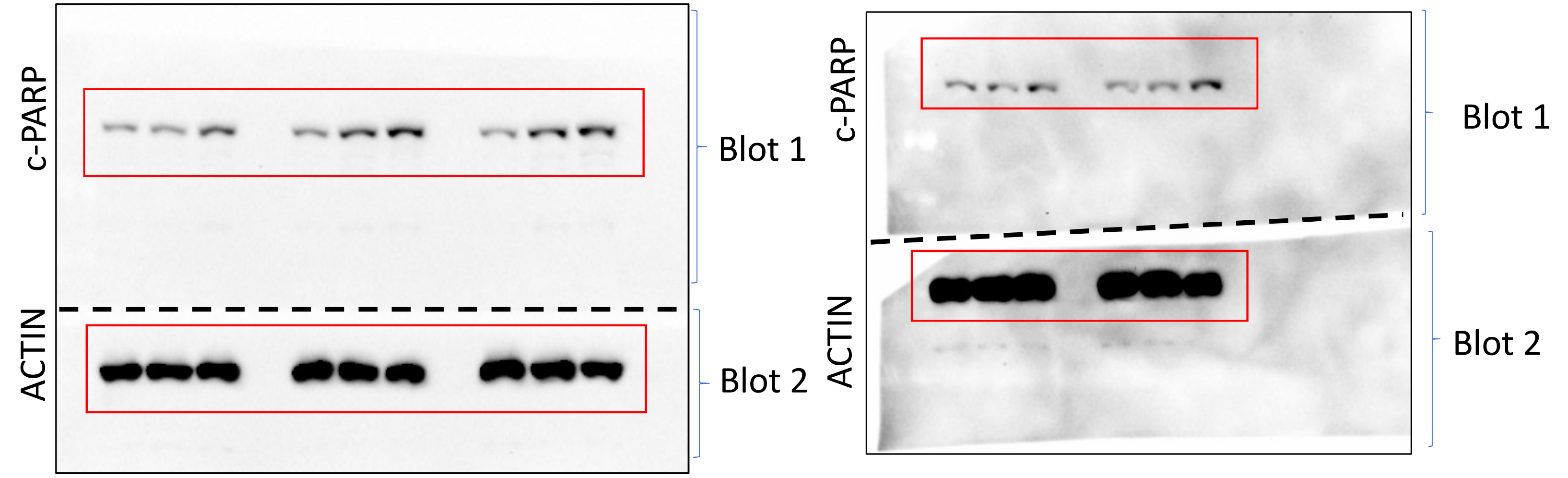

FIG S15b

Fig S4c.

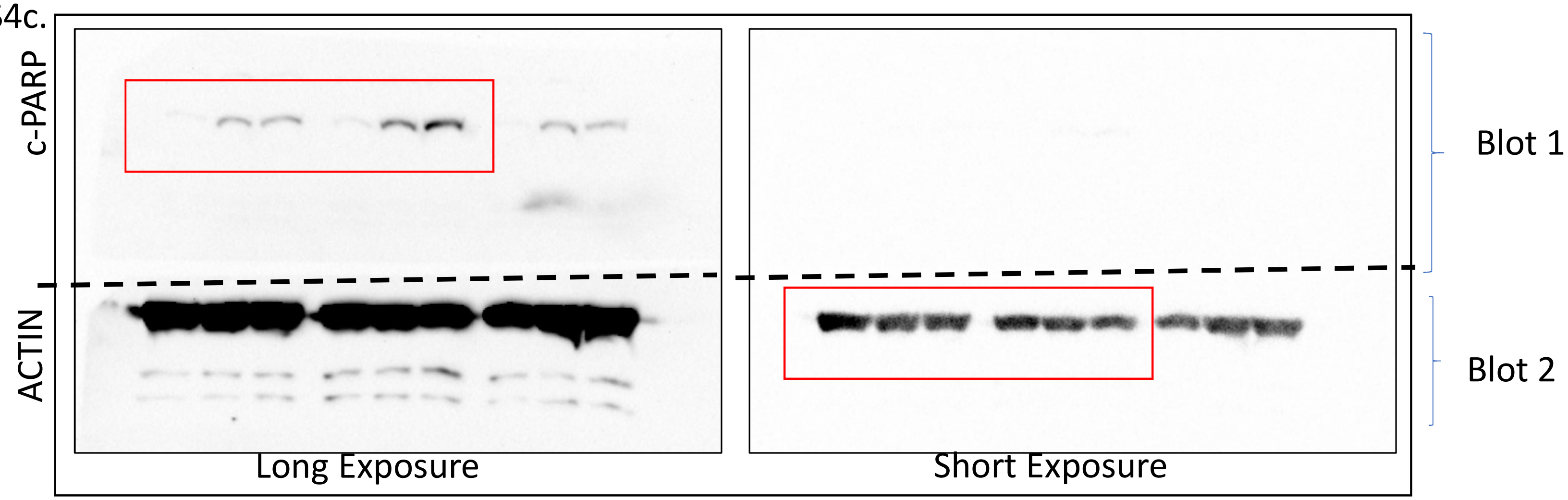

Fig S4d.

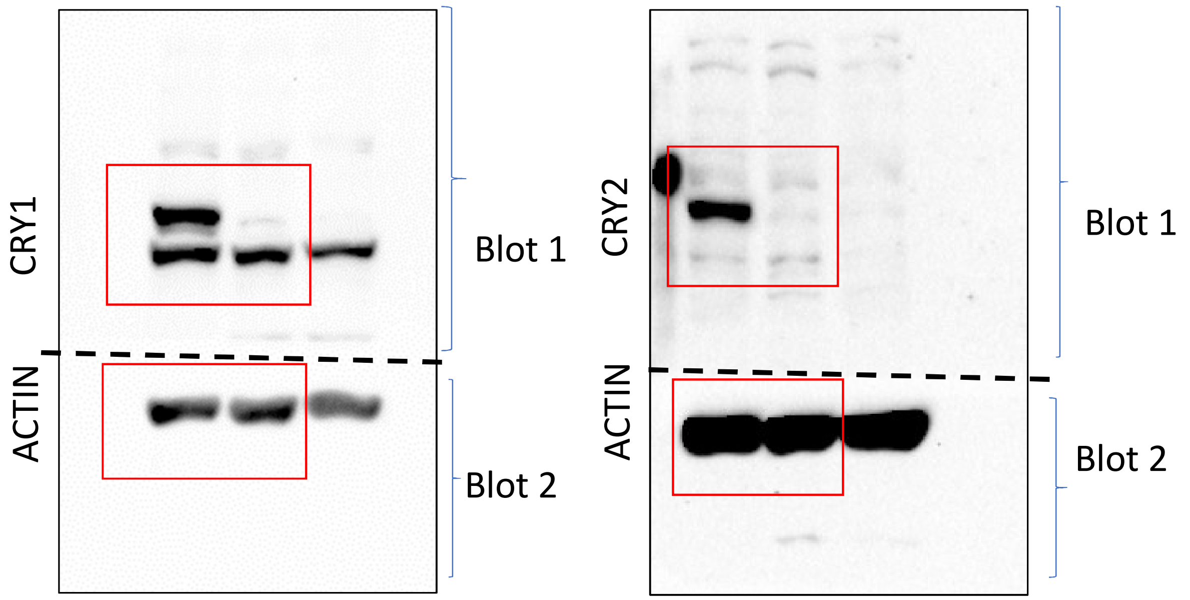

FIG S16a

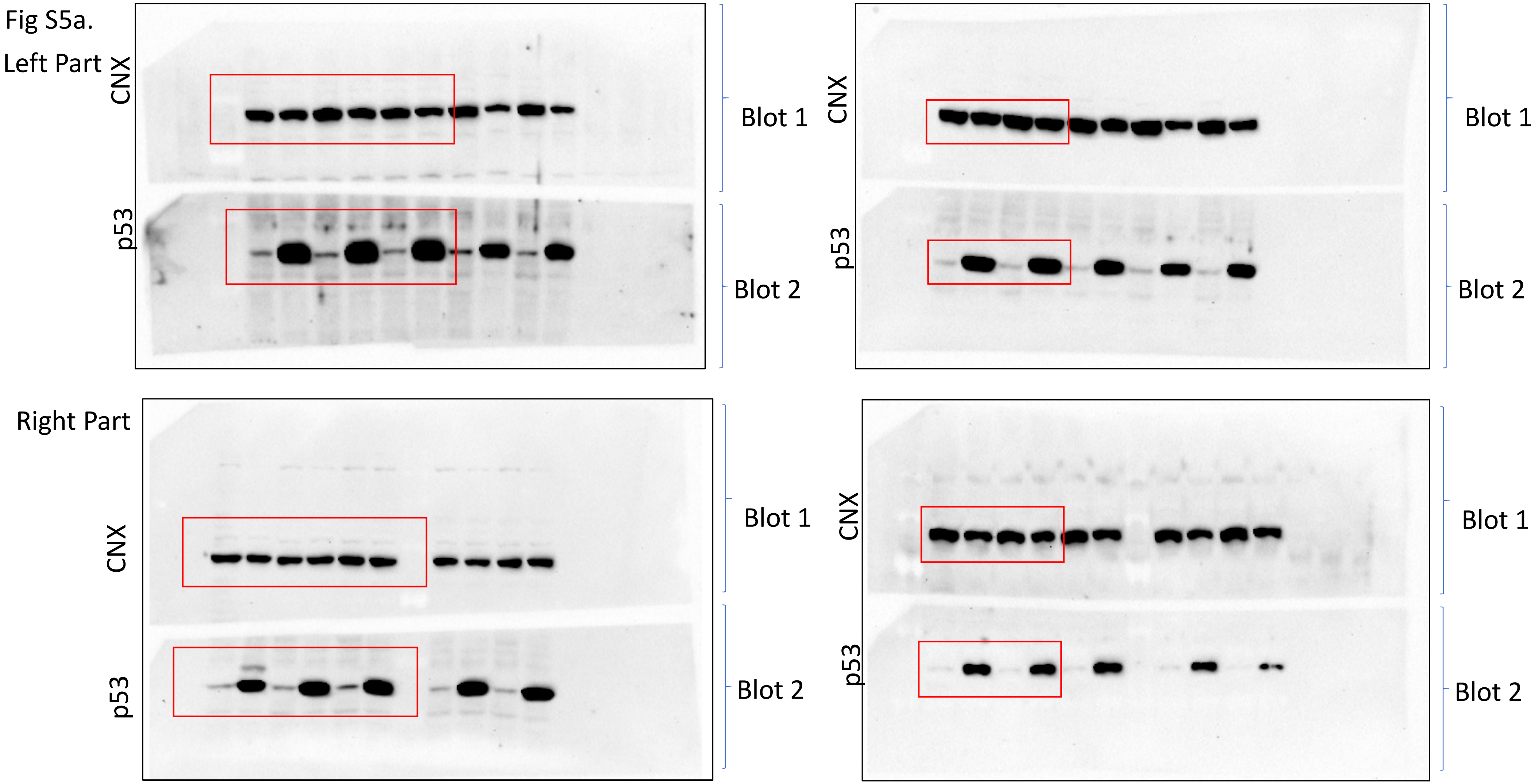
Fig S5a.
Left Part
CNX
p53
Blot 1
Blot 2
CNX
p53
Blot 1
Blot 2
Right Part
CNX
p53
Blot 1
Blot 2
CNX
p53
Blot 1
Blot 2

FIG S16b

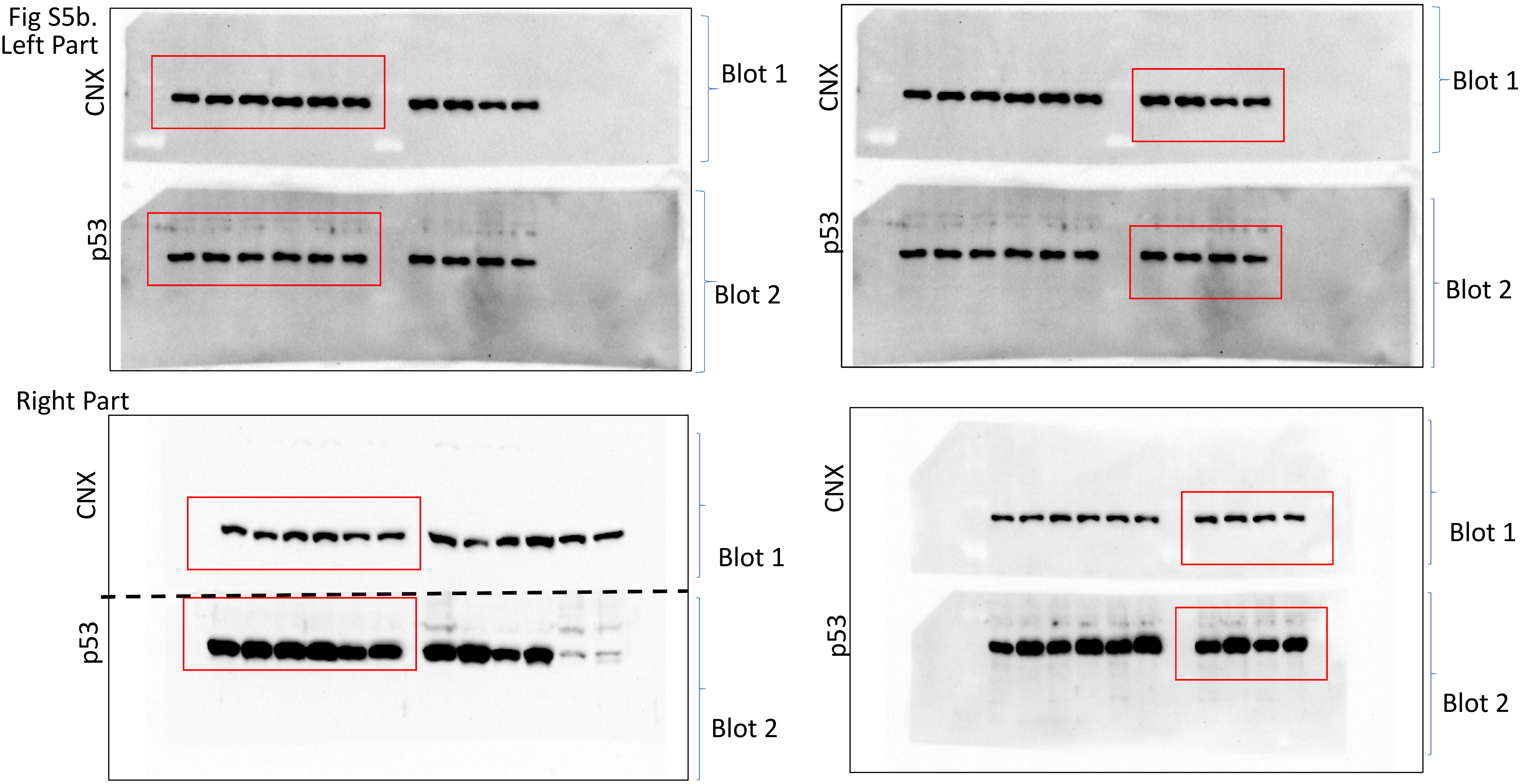

Fig S5b.
Left Part
CNX
p53
Blot 1
Blot 2
CNX
p53
Blot 1
Blot 2
Right Part
CNX
p53
Blot 1
Blot 2
CNX
p53
Blot 1
Blot 2

FIG S17

Fig S7.

MCF10A

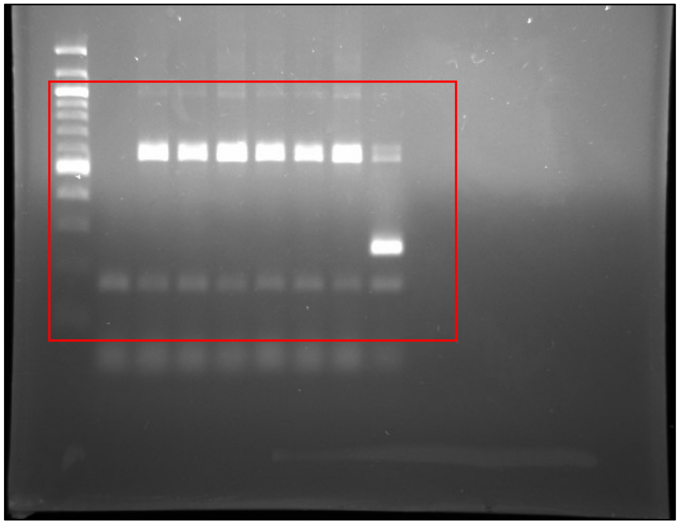

MDA-MB-231

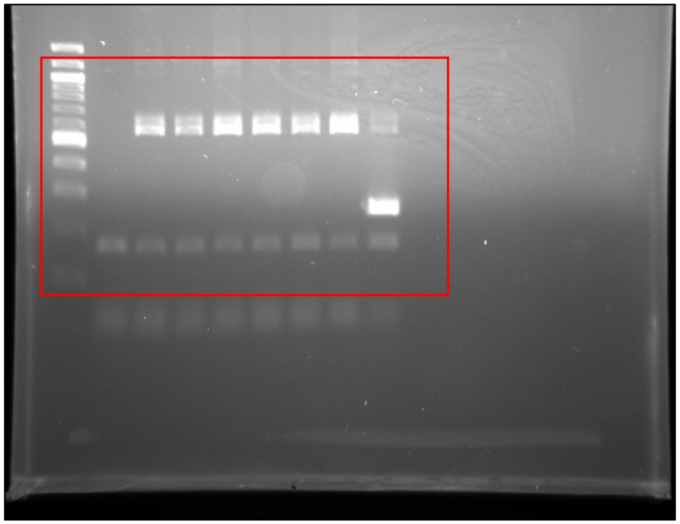

Supplement: Supplementary file 1 — Supplementary Information [file 41598_2018_34433_MOESM1_ESM.pdf]
